# An assessment of acute insecticide toxicity loading (AITL) of chemical pesticides used on agricultural land in the United States

**DOI:** 10.1371/journal.pone.0220029

**Published:** 2019-08-06

**Authors:** Michael DiBartolomeis, Susan Kegley, Pierre Mineau, Rosemarie Radford, Kendra Klein

**Affiliations:** 1 Toxicology Research International, Haiku, Hawaii, United States of America; 2 Pesticide Research Institute, Inc., Santa Rosa, California, United States of America; 3 Department of Biology, Carleton University, Ottawa, Ontario, Canada; 4 Friends of the Earth US, Berkeley, California, United States of America; University of California San Diego, UNITED STATES

## Abstract

We present a method for calculating the Acute Insecticide Toxicity Loading (AITL) on US agricultural lands and surrounding areas and an assessment of the changes in AITL from 1992 through 2014. The AITL method accounts for the total mass of insecticides used in the US, acute toxicity to insects using honey bee contact and oral LD_50_ as reference values for arthropod toxicity, and the environmental persistence of the pesticides. This screening analysis shows that the types of synthetic insecticides applied to agricultural lands have fundamentally shifted over the last two decades from predominantly organophosphorus and N-methyl carbamate pesticides to a mix dominated by neonicotinoids and pyrethroids. The neonicotinoids are generally applied to US agricultural land at lower application rates per acre; however, they are considerably more toxic to insects and generally persist longer in the environment. We found a 48- and 4-fold increase in AITL from 1992 to 2014 for oral and contact toxicity, respectively. Neonicotinoids are primarily responsible for this increase, representing between 61 to nearly 99 percent of the total toxicity loading in 2014. The crops most responsible for the increase in AITL are corn and soybeans, with particularly large increases in relative soybean contributions to AITL between 2010 and 2014. Oral exposures are of potentially greater concern because of the relatively higher toxicity (low LD_50_s) and greater likelihood of exposure from residues in pollen, nectar, guttation water, and other environmental media. Using AITL to assess oral toxicity by class of pesticide, the neonicotinoids accounted for nearly 92 percent of total AITL from 1992 to 2014. Chlorpyrifos, the fifth most widely used insecticide during this time contributed just 1.4 percent of total AITL based on oral LD_50_s. Although we use some simplifying assumptions, our screening analysis demonstrates an increase in pesticide toxicity loading over the past 26 years, which potentially threatens the health of honey bees and other pollinators and may contribute to declines in beneficial insect populations as well as insectivorous birds and other insect consumers.

## Introduction

Insects form the basis of the food web that sustains life on Earth. They are critical to ecosystem success, providing food for amphibians, fish, birds, reptiles, and mammals. Insects play a role in decomposing animal wastes and dead vegetation, recycling the nutrients in these materials and returning them to the soil. Insects also contribute to the agricultural production of crops that feed humankind, both as the primary pollinators of many plants and as natural controls of pest insects that feed on crops important to human survival. A diverse population of insects benefits agriculture by keeping a balance between predatory and pest insects and providing pollination services [1].

Insecticides targeting crop-damaging pests reduce both the number and diversity of insects in an ecosystem [2]. With conventional farming practices relying primarily on chemical insecticides for pest insect management, ecosystems comprising US agricultural lands are highly impacted through both direct effects on insects and direct and indirect effects on other species [3]. Although many members of the ecosystem may not be exposed to sufficient doses of insecticides to suffer acutely lethal poisonings, sublethal and indirect adverse effects have been demonstrated to occur [4].

### Insecticide use patterns in the US

**Fig 1:**
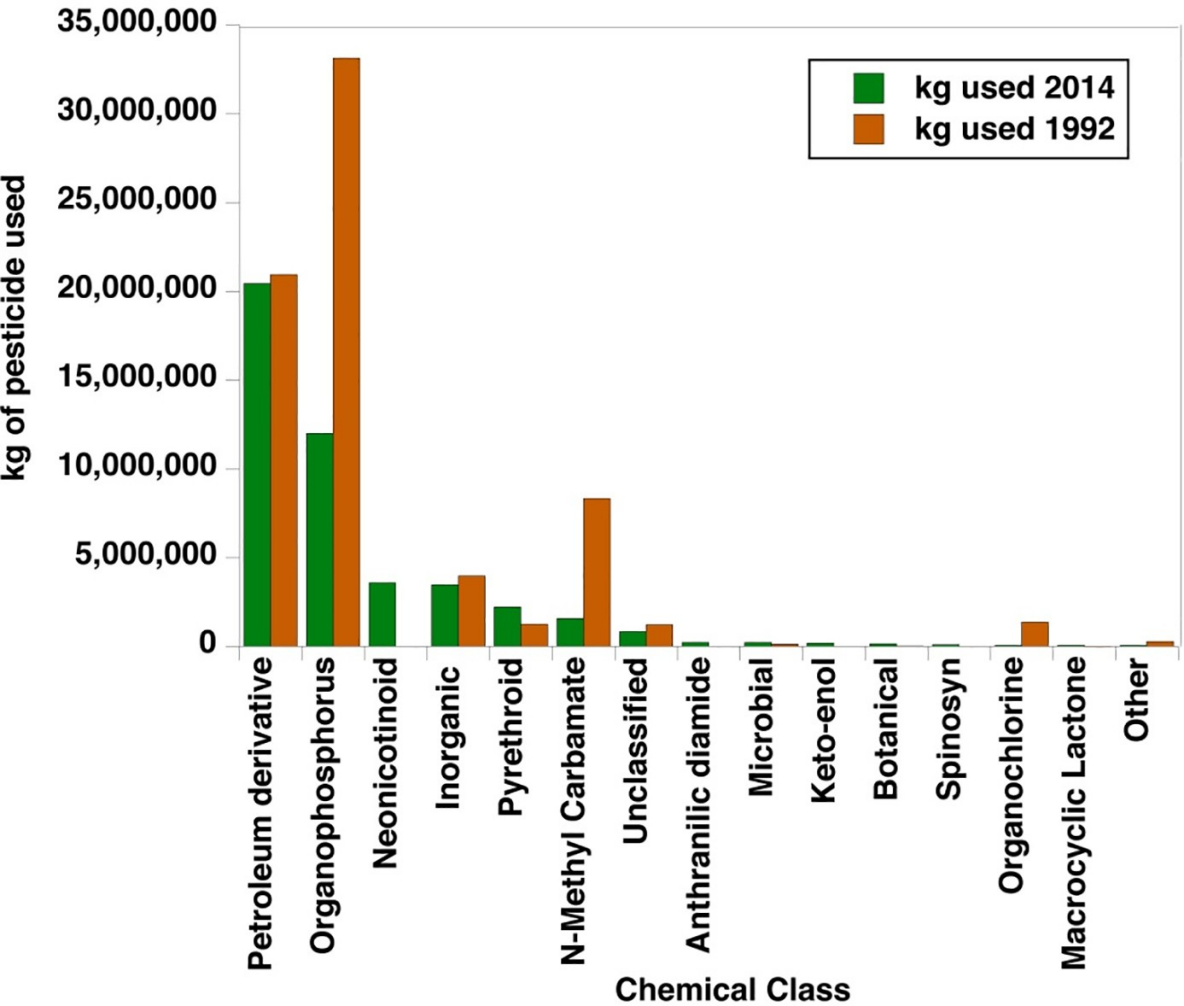
Change in use of insecticide chemical classes in the US (1992–2014). Data source: US Geological Survey pesticide use estimates for the US [5–7].

The types of synthetic insecticides applied to agricultural lands have fundamentally shifted over the last two decades from predominantly organophosphorus and N-methyl carbamate insecticides to substantially lower amounts of organophosphorus compounds along with a substantial increase in neonicotinoids and a modest increase in pyrethroids (Fig 1). Petroleum derivatives such as mineral oil and inorganics such as kaolin clay, lime-sulfur, cryolite, and borates remain as some of the primary lower-toxicity chemical classes of insecticides in current use, with little change over time.

These changes in use patterns reflect the outcome of US Environmental Protection Agency (US EPA) re-registration of pesticides mandated by the Food Quality Protection Act of 1996 and the development of new pesticide chemistries targeting different receptors in insect physiology to combat resistance in pest species [8]. These changes have almost certainly altered the toxicity landscape for insects. In general, systemic pesticides, in particular the neonicotinoids, are now one of the preferred or most readily available and economically efficient class of insecticides used in conventional agriculture practices in rotation with carbamate, pyrethroid, and organophosphorus-containing pesticide products, many of which are still registered for use in the US. The organophosphorus and N-methyl carbamate classes of pesticides are highly toxic to insects but are not especially persistent in the environment, with half-lives ranging from several days to several weeks [9, 10]. Neonicotinoids, like organophosphates and N-methyl carbamates, are neurotoxicants that target the central nervous system by binding to nicotinic acetylcholine receptors leading to overstimulation and paralysis. However, neonicotinoids generally pose lower acute hazards to mammals and greater toxicity to insects due to their differential binding abilities to invertebrate and vertebrate cholinergic receptors (Table 1) [11]. The nitro-substituted neonicotinoids, including imidacloprid, thiamethoxam, and clothianidin (which is also a metabolite of thiamethoxam), are the most frequently used neonicotinoids and tend to have measurably greater persistence than the organophosphorus, carbamate, and pyrethroid insecticides, with half-lives of 39 to 174 days in soils (see S1 Appendix for the source information of these data). In addition, the neonicotinoids exhibit higher water solubility, leading to greater exposure potential for insects consuming pollen, nectar, guttation water, or plant tissue or aquatic insects exposed to runoff containing these pesticides [12]. On the other hand, lipophilic chemicals would tend to accumulate more in the lipid components of pollen and bee bread [13].

**Table 1:** Top ten most acutely toxic insecticides to honey bees by the oral route.

Active Ingredient	Chemical Class	Environmental Half-life (days) ^‡^	Honey Bee Oral LD50 (μg/bee)^†^	Mammalian LD50* (mg/kg)
Fipronil	Pyrazole	65	0.003	92 (II)
Imidacloprid	Neonicotinoid	174	0.0037	424 (III)
Thiamethoxam	Neonicotinoid	39	0.005	1,563 (III)
Abamectin	Macrolide	1	0.0063	11 (I)
Clothianidin	Neonicotinoid	121	0.0079	>5,000 (IV)
Deltamethrin	Pyrethroid	21	0.011	>5,000 (IV)
Monocrotophos	Organophosphorous	30	0.02	23 (I)
Mevinphos	Organophosphorous	1	0.027	2.2–12 (I)
Beta-Cyfluthrin	Pyrethroid	13	0.035	11 (I)
Dinotefuran	Neonicotinoid	75	0.04	2,000 (III)

^**‡**^ Source of half-life data provided in S1 Appendix, and is predominantly obtained from field testing and/or soil persistence.

† All oral LD50s for these active ingredients are considered “highly toxic” (<2 μg/bee) using US Environmental Protection Agency’s criteria.

* Acute mammalian toxicity category is given in parentheses: I = Highly Toxic; II = Moderately Toxic; III = Slightly Toxic; IV = Not Acutely Toxic

Sources: Half-life data S1 Appendix, Honey bee LD_50_s S1 Appendix, and mammalian LD_50_s US Environmental Protection Agency.

Although the neonicotinoids are highly toxic to insects, their effects are not confined to insects. For example, recent analyses indicate that insectivorous bird declines observed in the Netherlands and France appear to be associated with the use of neonicotinoid insecticides in the field or as seed treatments [14, 15]. Another review of the direct and indirect ecosystem effects of insecticides linked impaired growth in fish to reductions in invertebrate prey due to imidacloprid and fipronil use and linked reductions in lizard species to the effects of fipronil on termite prey [3]. Surface waters in agricultural areas have been shown to contain concentrations of neonicotinoids that exceed acute and chronic “invertebrate aquatic life benchmarks” and toxicity thresholds (e.g., no observed effect concentrations or NOEC) for aquatic life [16, 17].

Long-term pest control often suffers from pesticide application since beneficial predatory insects that consume pest insects are susceptible to insecticide exposure and often not as quick to rebound [18–20]. Prophylactic use of neonicotinoids as seed treatments in corn, soy, and other crops has risen in recent years; research has shown that this use has potentially damaged predatory beneficial insect populations and disrupted integrated pest management (IPM) programs [21].

### Honey bees as an indicator species of ecotoxicity

Honey bees are the most well studied indicator of insect health in US agricultural lands and surrounding areas. Because they are economically important for crop pollination, honey production, and wild plant pollination, the National Agricultural Statistics Service (NASS) tracks colony counts and honey production in the US [22]. The honey bee (*Apis mellifera*) is generally considered to be relatively sensitive to pesticides when compared to other bee species [23] and has historically been used as an indicator for ecotoxicological testing. However, there has also been some concern that the honey bee is not a good indicator for other bees or other beneficial insects because of species differences in autecology and sensitivity [24]. Information is being developed on the toxicity of insecticides to pollinators other than honey bees, notably bumble bees (*Bombus* species) and several solitary bee species. However, to date, data are only available for a small proportion of active ingredients, and tests have not been standardized. Heard *et al*. developed a “standardized” toxicity test system to compare the relative sensitivity between bee species in terms of a pesticide’s toxic potency and the time needed for the onset of toxicity [24]. Although there were significant inter-species differences that varied through time, overall, the magnitude of these differences was generally within an acceptable two-fold range.

A recent meta-analysis of paired toxicity data from the same sources demonstrated a high variability of sensitivity among bee species (Max/Min ratio from 0.001 to 2085.7) [23]. However, an extrapolation factor of 10 applied to honey bee toxicity endpoints was sufficiently protective in 95 percent of cases, and the honey bee tended (as shown by a median value of ratios) to be slightly more sensitive than the paired test species. Sanchez-Bayo and Goka regressed *Bombus* LD_50_ values against *Apis* LD_50_ values and concluded that the susceptibility of both genera was similar when exposed by the oral route [25]. However, the honey bee was found to be more sensitive than bumble bees by the contact route even after correcting for weight. It is clear that the susceptibility of any one insect species could be substantially different from another.

In our work, we use honey bee toxicity as an indicator for other bees and beneficial insects in US agricultural land because the available data appear to demonstrate that the honey bee is sensitive to the toxicity of chemical pesticides and has the most comprehensive data set available for insects. Until more data on other insects become available, the use of the honey bee as an indicator for other species is a reasonable approach to show how insecticide toxicity loadings have changed over time.

The toxicity database on honey bees is compiled from test results submitted by pesticide manufacturers (“registrants”), academic researchers, and other independent research institutes. In order to register (license) a pesticide product in the US, applicants for registration must satisfy several criteria specified in the Federal Insecticide, Fungicide, and Rodenticide Act (FIFRA) including but not limited to the product’s toxicity in a variety of biological systems, its fate and impact on the environment, and for certain pesticide products, proof of its performance (efficacy) [26]. Acute lethality (LD_50_) testing in honey bees is required under FIFRA, however, field tests are only required on a rarely invoked case-by-case basis. Despite these limitations and data gaps, the acute toxicity data base (LD_50_s) for honey bees is sufficient to allow for a comparative screening analysis of acute insecticide toxicity loading in the environment.

### Assessing the acute toxicity loading of insecticides on US agricultural land and surrounding areas

An assessment of changes in the types and amounts of insecticides used over time and consideration of potential environmental impacts is illuminating. We present here a method for assessing the Acute Insecticide Toxicity Loading (AITL) on US agricultural lands and surrounding areas for terrestrial insects using toxicity data for the honey bee as an indicator for all arthropods. We developed the AITL method in order to allow for a screening level analysis of the historical loading of pesticides onto agricultural land and surrounding areas over the past two decades and as a metric for evaluating their *potential* for causing detrimental impacts on beneficial insects such as pollinators and other non-target species.

Recently, researchers in Great Britain published a comparable method [27]. In this work, the authors investigated the occurrence of changes in the mass of pesticides used, the area sprayed, and the total number of honey bees that could potentially be killed in Great Britain in the period covering 1990 to 2015. Our AITL analysis is an internally consistent estimate, which accounts for the total mass of toxic pesticides applied in the US and to specific crops and the acute toxicity of each pesticide to the honey bee. However, unlike the previously published method, the AITL also accounts for pesticide persistence in the environment (i.e. dissipation rate in field). The AITL values were calculated by chemical class, by individual chemical for the top chemicals contributing to the loading, and by crop groups as defined in the US Geological Survey (USGS) pesticide use database [5–7].

We believe the incorporation of persistence (e.g., as measured by half-life in the field and/or soil) of pesticides in this analysis is crucial to understanding the long-term and cumulative ecosystem toxicity beyond the initial pesticide application to a crop. For example, although organophosphorus insecticides are highly toxic to insects, they generally have half-lives less than 30 days and do not present a long-term hazard for insects. This characteristic allows for the mitigation of the risk to pollinators through application timing that avoids periods of bloom. In contrast, neonicotinoid residues from seed treatments may be found in the soil for months or even years after planting [12, 28]. For example, neonicotinoid insecticides applied on coated seeds [18], mature citrus trees [29], or as soil drenches [12] on annual crops have been found to be effective at killing insects more than 50 days from treatment or planting of treated seeds. For perennial crops such as trees and vines, insecticidal efficacy can last for months up to a few years under certain conditions [30].

To account for persistence, similar to the methods used to estimate the dose of a drug [31], we estimated pesticide loading to the US agricultural land and surrounding areas as the area under the curve of degradation/dissipation of pesticides over time. We assumed typical first-order kinetics, which is used by US EPA to estimate pesticide degradation (see Methods).

The AITL analysis does not account for toxicity effects other than lethality or for synergistic effects from co-application of different active ingredients. The analysis also does not provide specific information on actual exposures experienced by insects in the environment nor on the timing and mode of pesticide application or the dissipation of the pesticide into the environment. Therefore, the AITL is not a standard risk assessment method (i.e., estimating the probability of harm) based on quantified actual or predicted exposure.

We propose that the AITL could be used as a screening tool by providing year-to-year comparison of toxicity loading over time, measuring change in the potential toxicity of chemicals released into the environment, predicting potential impacts of new insecticides being considered for registration, and for surveying insecticide use and impacts on agricultural land. In this paper, we apply our AITL methodology to analyze how acute toxicity loading for insects in US agricultural land and surrounding areas changed between 1992 and 2014 and to identify the pesticidal chemical classes, the specific chemical active ingredients, and the crops that contributed most to these changes.

## Methods

### Pesticide use data

Pesticide use data were obtained from USGS and include foliar, soil, and seed treatment uses of pesticides [5–7] from 1992–2014. USGS reports agricultural pesticide use at the county level, which are based on farm surveys of pesticide use and estimates of harvested crop acres. Data collected after 2014 were not included, since the data collection methods no longer incorporate pesticides used as seed treatments. USGS developed two estimates: the “EPest High” estimate that interpolated for missing data and the “EPest Low” estimate which simply assumed zero use if data were missing. We used the EPest High data for our assessment because it provides a more complete and realistic quantitative description of pesticide use in the US. It should be noted that data are missing from this data set for insecticides used on soybean crops between 1998 and 2003 because this question was omitted in grower surveys (USGS, personal communication). Also, pesticides for which no environmental half-life or either oral or contact honey bee LD_50_ values were available were not included in the analysis.

**Fig 2:**
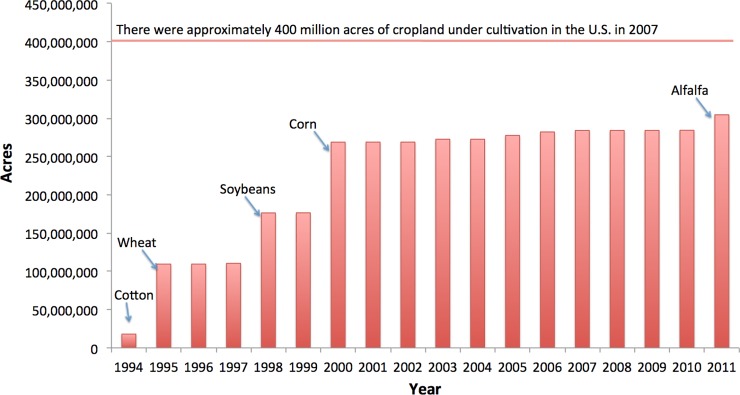
Crop acreage in the US on which neonicotinoid insecticides could legally be used based on 2007 data for acres planted. Data source: US Federal Register notices, US Environmental Protection Agency 1992–2017 [32].

Nationwide, data on acres treated with different pesticides do not exist for the time period in question, but approvals for new use of systemic insecticides on cropland can be tracked via tolerance decisions published in the Federal Register [32]. We determined acres that could legally be treated using the USDA National Agricultural Statistics Service acres planted data from the Census of Agriculture (Fig 2) [22]. By this measure, the acres of US cropland that could be treated with neonicotinoids have increased every year, with large increases in potential use when approvals were obtained for high-acreage commodity crops like corn, soybeans, cotton, wheat, and alfalfa. As noted previously, seed coatings comprise the largest contribution to increasing use [33], although studies do not consistently demonstrate economic benefits to farmers from insecticidal seed treatments [34].

### Toxicity and environmental persistence data

In calculating the AITL, we used honey bee contact (often referred to in the literature as topical) and oral LD_50_ values as an indicator for pesticide toxicity to insects, referred to as AITL_C_ and AITL_O_, respectively. Honey bee LD_50_ values for registered insecticides were obtained from a variety of sources and are provided in the supporting materials that accompany this publication (S1 Appendix). The database for LD_50_s is a compilation of data publicly available from several databases managed by government agencies, academic institutions, and independent research institutes worldwide. Values generated for the technical grade active ingredient were used preferentially, although data obtained with formulations were used if technical grade active ingredient LD_50_s were not available. Toxic degradates were included in the analysis if the degradate was also a registered pesticide and the AITL_C_ of the parent pesticide was greater than or equal to (≥) 0.1 percent of the total AITL_C_ for the period 1992–2014. In practice, this criterion excluded all but clothianidin produced from the degradation of thiamethoxam, where 35.6 percent of applied thiamethoxam degrades to clothianidin within 90 days [35]. This portion of clothianidin was analyzed separately for source clarity.

Excluded from the analysis were known low acute toxicity inorganic pesticides (e.g., cryolite, sulfur), low acute toxicity petroleum derivatives (e.g., mineral oil), microbial pesticides (e.g., *Bacillus thuringiensis*), and low-use (<5,000 kg over the time period 1992–2014) pesticides. The only high-use, potentially higher toxicity pesticide for which LD_50_ values could not be found is phostebupirim (tebupirimphos), which excluded it from the analysis [36]. A range of LD_50_ values for honey bees has been reported for some pesticide active ingredients, and for some we have concerns over the quality of the data. In order to consistently and comparably select LD_50_s to use in our analysis, we developed a set of explicit rules which we applied in the selection process (Table 2). These rules were used independently for both contact and oral toxicity values.

**Table 2:** Guidelines used in selecting LD_50_ values from multiple sources of data.

Rule Number	Available LD50 Data	Application
1	Single "exact" value reported	Used unmodified in analysis
2	Single value reported but qualified as "approximate" or "greater than" (>)	Used unmodified in analysis
3	Multiple "exact" values reported	Arithmetic mean of all values used in analysis unless the difference between the lowest and highest values was greater than 10-fold and then the geometric mean is used
4	Multiple values reported but all qualified as "greater than" (>)	Highest value used in analysis
5	Values reported but qualified as "less than" (<)	Not used in analysis

Aerobic half-lives for pesticide chemicals were obtained from several sources. The preferred source was the Pesticide Properties Database (PPDB) field half-life [37]. If a field half-life value was not available in the PPDB, we used the soil half-life from this database. If any half-life value for a chemical was not available from the PPDB database, the aerobic half-life from the California Department of Pesticide Regulation Status Reports for the Pesticide Contamination Prevention Act [9] was used.

### Acute toxicity loading for insects

Our approach provides a general measure of acute toxicity loading of insecticides on US agricultural land and surrounding areas, assuming insects are exposed to pesticides released to the environment through direct contact with contaminated surfaces, water, or food or through ingestion of contaminated food or water. Different insects will have different exposures depending on their habitat, behaviors, and food sources; however, across years, exposures for different types of insects will be comparable. However, as noted previously, this analysis does not include actual or estimated exposure doses, nor does it factor in timing and mode of pesticide application. Therefore, the AITL method would best be described as a screening analysis that can identify or predict potential environmental impacts.

Honey bee lethality is the measure of toxicity used to assess AITL. This analysis was developed for both contact toxicity (AITLc) and oral toxicity (AITLo). The AITL_C_ calculation provides the number of toxicity loading units (TLU) applied to a crop for each pesticide by dividing the mass of chemical applied (in μg) by the honey bee contact LD_50_ (in μg/bee) (the first term in Eq 1 below) to give the number of honey bee LD_50_’s released to the environment. This value is then modified by the half-life of the chemical (in days), assuming exposure continues as long as the chemical is present, with degradation governed by the half-life of the chemical and the dose expressed as the area under the curve of concentration versus time (second term in Eq 1). Because the AITL values obtained are on the order of 10^12^–10^18^, a scaling factor of 10^−15^ is included to scale the values for plotting the results. The same method of calculation is applied for AITLo (Eq 2).

$${AITL}_{C}=\left(\frac{\mu g\;pesticide}{Honey\;bee\;contact{\;LD}_{50}\;(\mu g/bee)}\right)\times \frac{half–life\;(days)}{\ln\;2}\times 10^{-15}(scaling\;factor)(in\;{LD}_{50}–days)$$ (1)

$${AITL}_{O}=\left(\frac{\mu g\;pesticide}{Honey\;bee\;oral\;{LD}_{50}\;(\mu g/bee)}\right)\times \frac{half–life\;(days)}{\ln\;2}\times 10^{-15}(scaling\;factor)(in\;{LD}_{50}–days)$$ (2)

Toxic degradates are known for some pesticide active ingredients. However, because environmental half-lives were not available for most of these compounds they were not included in the analysis. Those degradates with known toxicity (e.g., malaoxon, the degradate of malathion) might contribute to overall acute toxicity, although we determined that most known degradates would contribute only a negligible amount to the overall toxicity loading of the parent compound. The one exception as noted previously is clothianidin, which is a metabolite of thiamethoxam; our analysis accounts for this conversion in the environment because it contributes a measurable level of toxicity relative to the parent compound.

**Fig 3:**
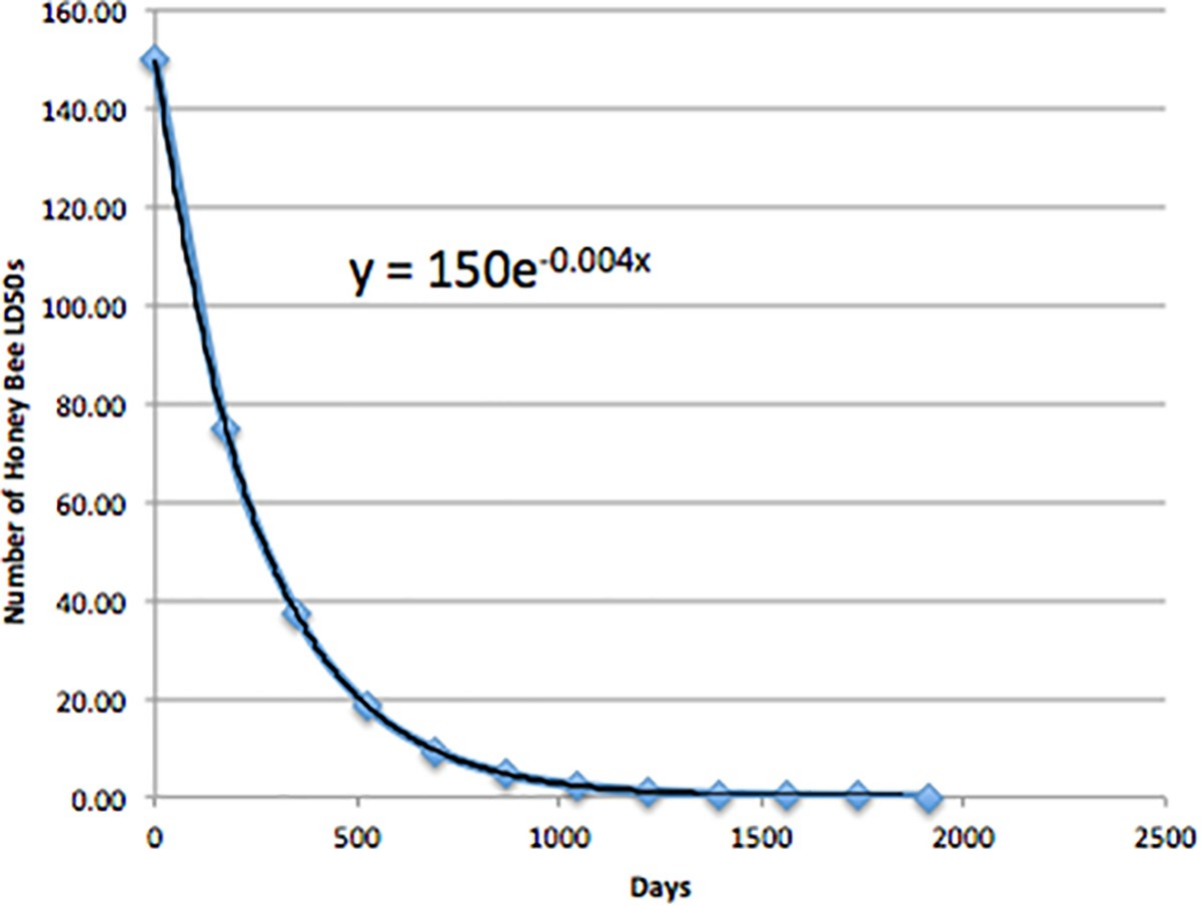
Theoretical degradation curve for imidacloprid following first-order kinetics with a half-life of 174 days.

We estimated pesticide loading on agricultural land and surrounding areas as the area under the curve of degradation/dissipation of pesticides over time, assuming typical first-order kinetics, as recommended by US EPA in its guidance [38]. While degradation rates vary depending on a number of factors, the first-order assumption is widely used for estimating pesticide concentrations in the environment over time, and this appears to be an appropriate assumption for the neonicotinoid insecticides [39, 40]. An example theoretical degradation curve for imidacloprid, with a half-life of 174 days, is shown in Fig 3. In this example, on Day Zero (application day), the available dose is 150 honey bee LD_50_s. On Day One, 149 honey bee LD_50_s still remain, with the potential for concomitant toxic effects to insects. On Day 174, 75 honey bee LD_50_s remain in the environment. Ninety-seven percent of the imidacloprid is degraded at five half-lives (870 days or 2.4 years). The total integrated environmental toxicity loading level over time can be calculated as the area under the curve. Therefore, we define AITL as the area under the curve in number of honey bee LD_50_-days, representing the total exposure potential for arthropods (both terrestrial and aquatic) over the degradation period.

For pesticides used as seed treatments, our analysis assumes that insect exposure from contact with treated crops would include dust drift to field-side plants during seed planting (which can be considerable) resulting in both contact and oral exposure, and oral exposure from consuming pollen, nectar, guttation droplets, or plant tissue from the treated crop [12]. In addition, application of the seeds to soil would result in exposure of the soil entomofauna and migration to waterways would result in exposures for aquatic insects. This is a simplifying assumption, which may or may not overestimate actual insecticide doses received by honey bees and other beneficial insects from seed treatments, depending on the specific circumstances. Based on a “residue per unit dose” estimation, it appears that seeding results in higher contamination of insects than an equivalent spray application but, due to the lower per hectare (or acre) rates of application for seed treatments, a comparable level of contamination in non-target arthropods can be expected [41]. Because the AITL is intended to be used as a screening level assessment for comparative and surveillance purposes, the inclusion of seed treatment applications is a reasonable approach. Further refinement of this method or other analyses would be required before making policy or regulatory decisions based on seed insecticide treatments alone.

## Results

### AITL calculations by chemical class

A comparison of AITLs calculated for different pesticide groupings demonstrates that insecticides contribute nearly 100 percent of the acute toxicity loading on honey bees and other beneficial insects of pesticides applied to agricultural land and surrounding areas in the US compared to herbicides, fungicides, and others (results not shown). Based on these preliminary calculations, we determined that the insecticides as a class represent the primary acute toxicity loading to insects in the environment. Therefore, no further analysis was conducted on the other pesticide groups.

#### Acute contact toxicity

**Fig 4:**
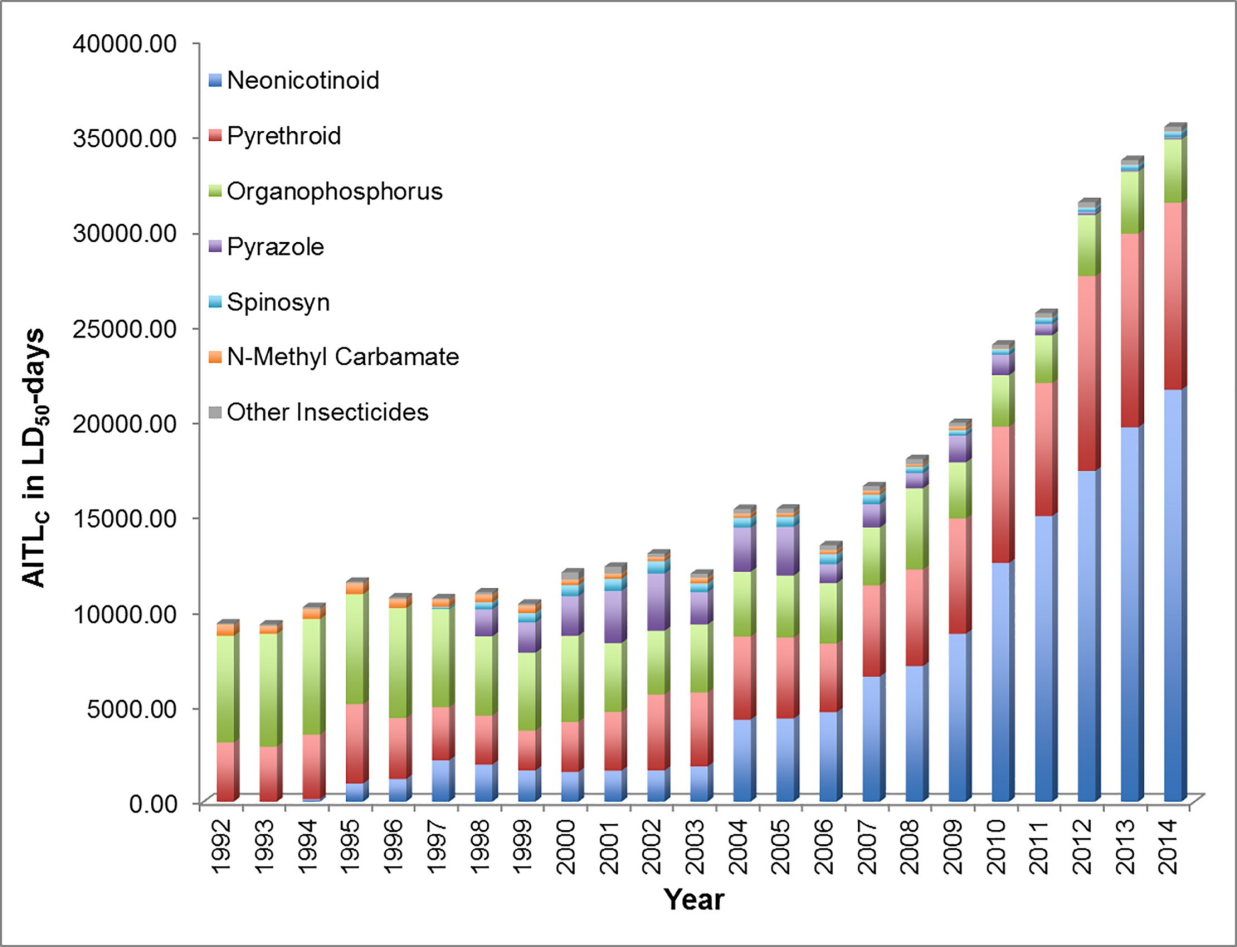
Contact acute insecticide toxicity loading (AITL_C_) by chemical class, 1992–2014.

AITL values were calculated for insecticidal active ingredients comprising several chemical classes for both acute contact (AITL_C_) and acute oral (AITL_O_) toxicity on agricultural land and surrounding areas in the US. Fig 4 presents the relative AITL_C_ values from 1992 to 2014 for six chemical classes as well as a miscellaneous category for contact acute toxicity (LD_50_s). From 1992, the first year included in our assessment, to 2014, the acute toxicity loading of pesticides in US agricultural land and surrounding areas based on AITL_C_ increased by 3.8-fold.

In the first decade of analysis, between 1992 and 2003, the AITL_C_ is the result of predominantly four classes of chemicals, the organophosphorus (43.4 percent on average), pyrethroid (28.5 percent on average), pyrazole (9.4 percent on average), and neonicotinoid (11.1 percent on average) insecticides. Although neonicotinoids had been introduced in 1994, our analysis indicates that the relative loading of this group of insecticides into the environment began to increase dramatically starting in about 2004 when the relative loading of the organophosphorus insecticides began to decrease. In 2004, the relative contribution of the neonicotinoids (27.8 percent) based on AITL_C_ surpassed that of the organophosphorus insecticides (22.0 percent) for the first time. By 2014, the relative contribution of neonicotinoids on the environmental toxicity loading via contact was 6.5 times greater than that of the organophosphorus insecticides. Pyrethroid insecticides contributed to the overall AITL_C_ relatively consistently from 1992 to 2014 (28.5 percent on average, range of 26.5 to 36.1 percent). Pyrazole insecticides (fipronil) contributed a smaller proportion of overall acute contact toxicity loading between 1992 and 2014 (6.2 percent) with the largest contribution occurring in a 12-year span from 1998 to 2010 (11.7 percent on average, range of 4.4 to 23.0 percent)[42], when its use on corn was cancelled [42]. The other insecticide classes analyzed contributed relatively small amounts to the overall AITL_C_ of insecticide use on the environment.

#### Acute oral toxicity

**Fig 5:**
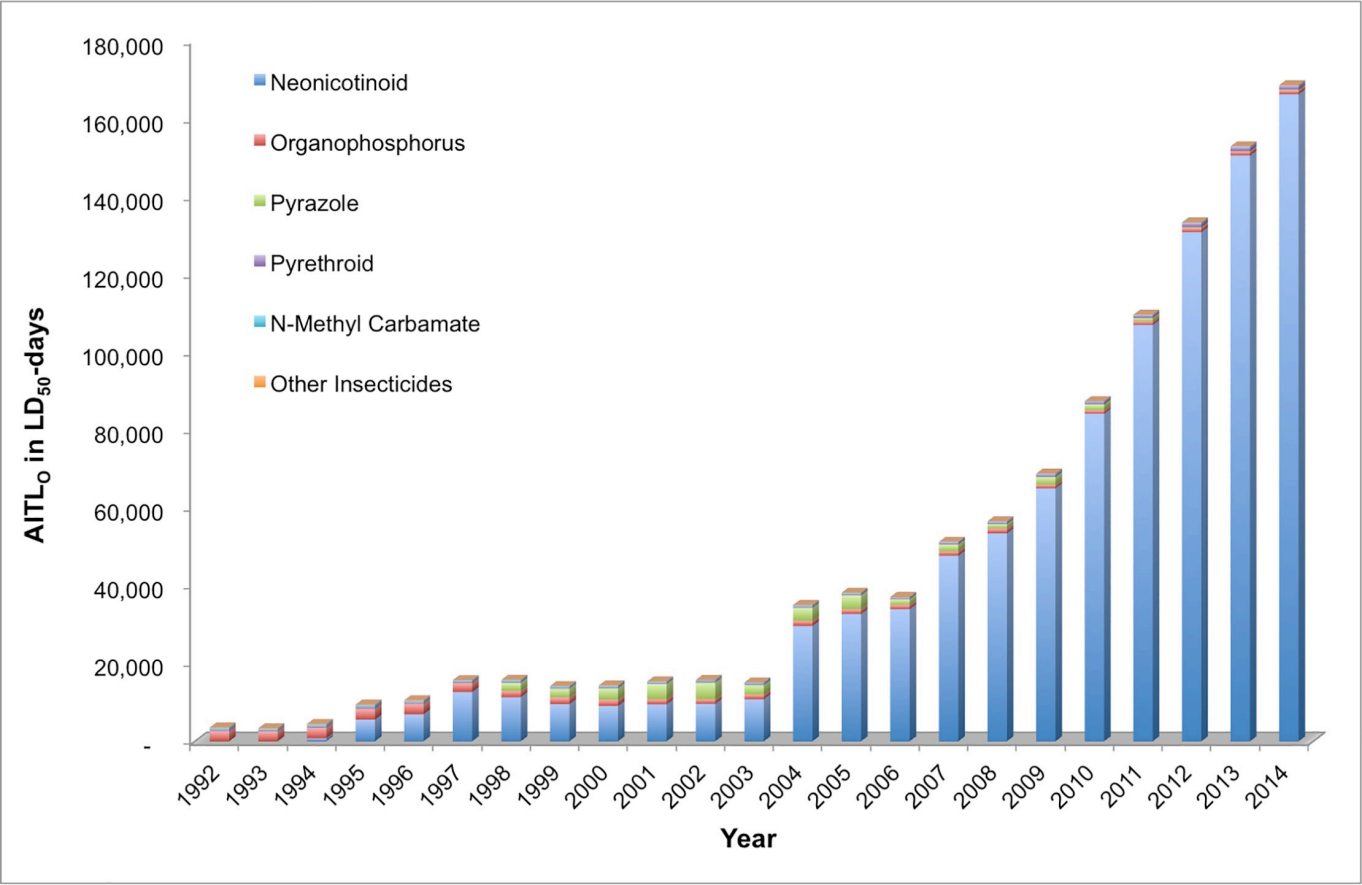
Oral acute insecticide toxicity loading (AITL_O_) by chemical class, 1992–2014.

On the basis of the acute oral toxicity loading (AITL_O_), the acute toxicity loading of insecticides in agricultural land and surrounding areas in the US was 48 times higher in 2014 compared to 1992. The AITL_O_ shows a vastly different trend in terms of relative chemical classes over the same 23 year time period compared to AITL_C_ (Fig 5). Although the organophosphorus insecticides comprised the majority of the acute toxicity loading between 1992 and 1994 (69 percent on average), from 1995 to 2014 the neonicotinoids comprise the majority (greater than 55 percent) of the overall AITL_O_ on the environment. The pyrazoles contributed on average 7 percent of the total AITL_O_ between 1998 and 2010 (range of 27 percent in 2002 to 1.7 percent in 2010), which is consistent with the analysis for acute contact toxicity (Fig 4). The relatively greater potential impact of the neonicotinoids on the environment based on the oral toxicity data is due to the relatively long environmental persistence of these chemicals and their high level of toxicity (i.e., relatively low LD_50_s) to honey bees and other insects via the oral route (Table 1).

#### Overall toxicity

In terms of absolute toxicity loading, the combined AITL_C_ for all chemical classes for acute contact toxicity increased by a factor of about 3.9 between 1992 and 2014 with the neonicotinoids contributing 60 percent of the total toxicity loading in 2014. However, the potential impact of the neonicotinoids is far more dramatic when looking at the absolute toxicity loading of all classes of insecticides based on the oral route of exposure. As noted above, the combined AITL_O_ for acute oral toxicity from all classes of insecticides increased by 48-fold from 1992 to 2014, with the neonicotinoids representing nearly 99 percent of the total acute oral toxicity loading in 2014.

### AITL calculations for active ingredients

**Fig 6:**
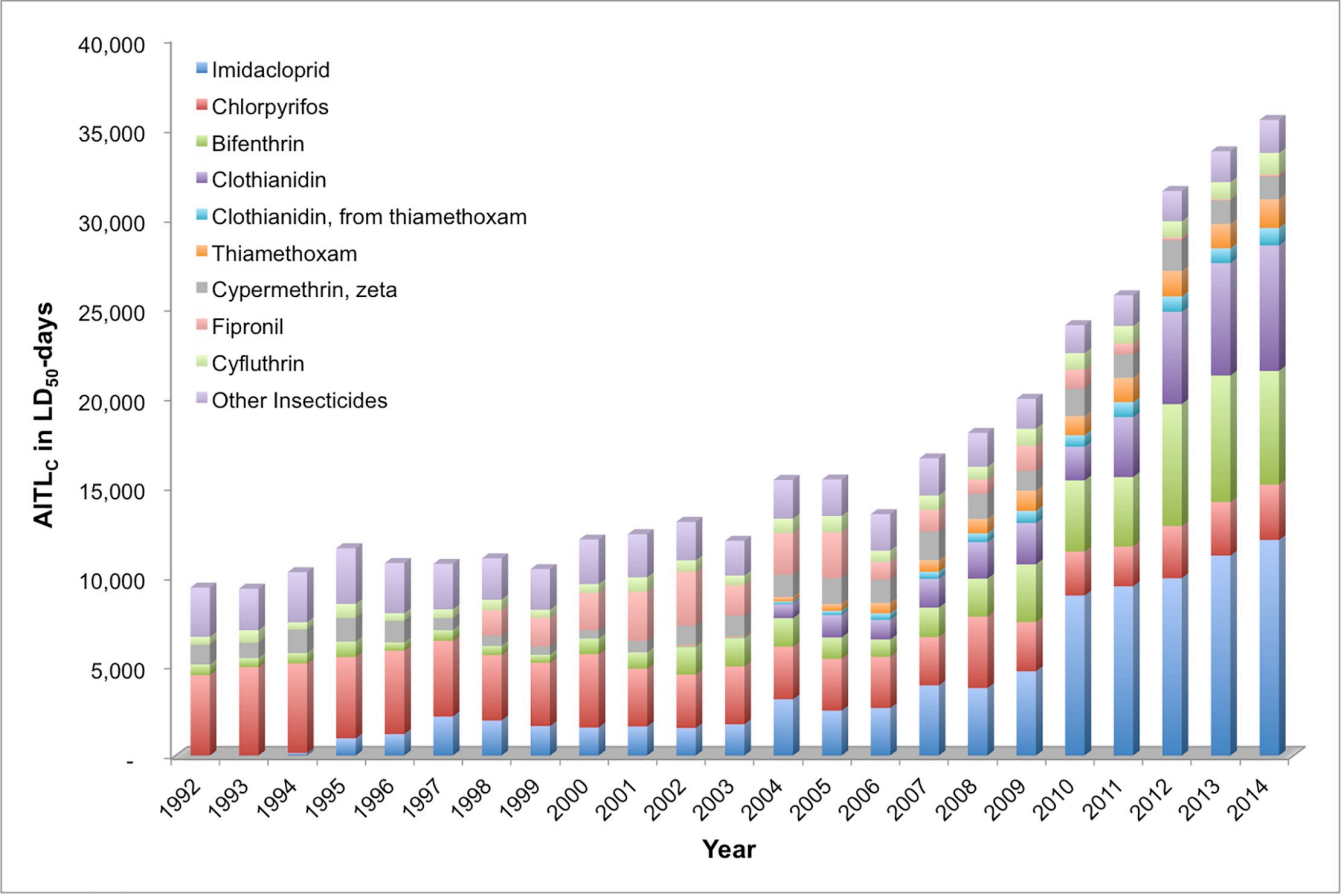
Contact acute insecticide toxicity loading (AITL_C_) by active ingredient, 1992–2014.

**Fig 7:**
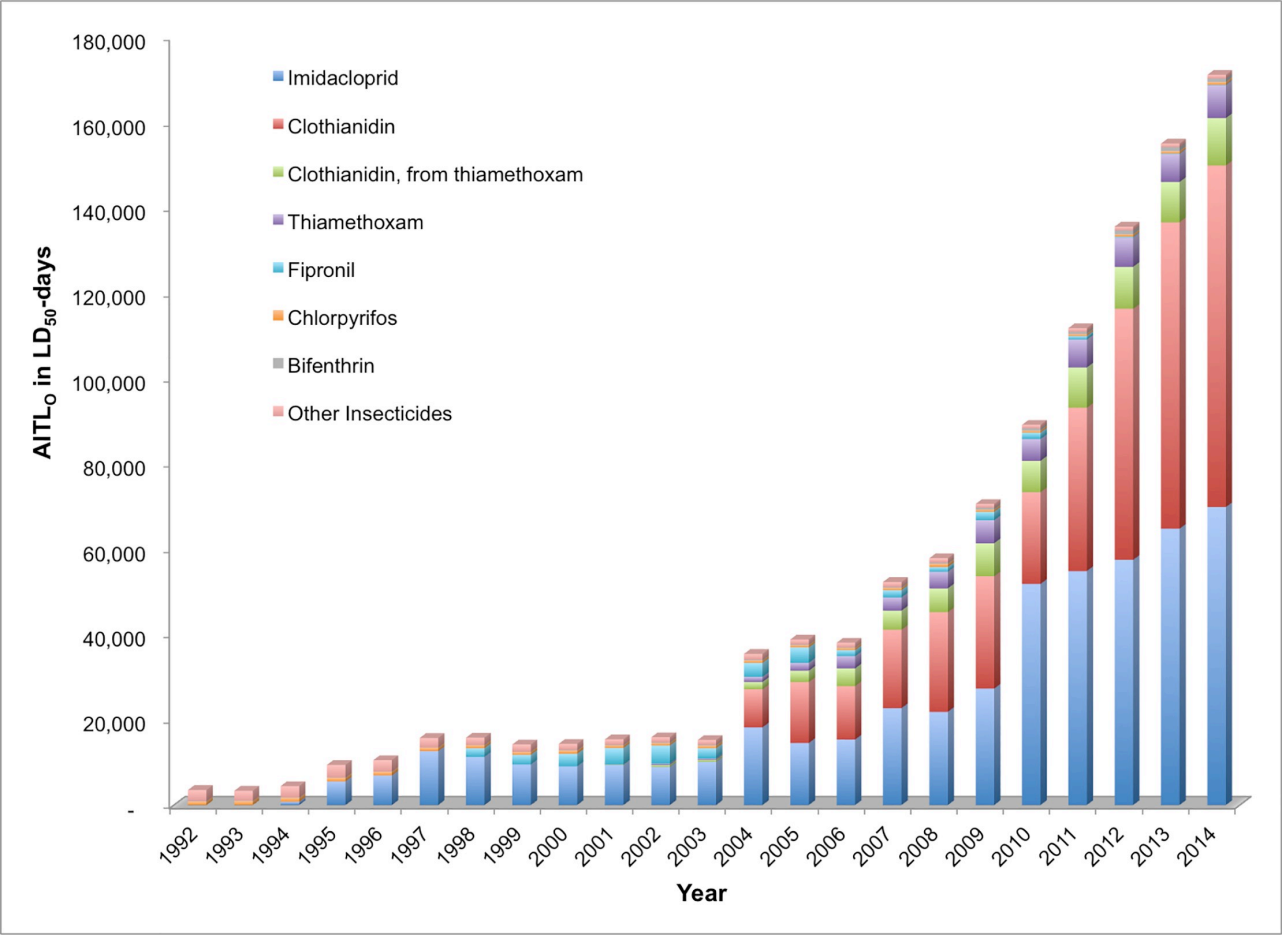
Oral acute insecticide toxicity loading (AITL_O_) by active ingredient, 1992–2014.

In order to determine which active ingredients contributed the majority of acute toxicity loading on agricultural land and surrounding areas in the US between 1992 and 2014, we calculated AITLs for individual chemicals representing the most toxic, persistent, and heavily used active ingredients in several chemical classes. AITL_C_ and AITL_O_ calculations for individual chemicals are presented in Figs 6 and 7, respectively.

#### Acute contact toxicity

With respect to AITL_C_ from 1992 to 2014, imidacloprid (20.0 percent) and chlorpyrifos (18.6 percent) comprise the two individual active ingredients with the most potential impact (Fig 6) over the 23-year period. Other individual insecticide active ingredients contributing a large proportion to the overall acute contact toxicity loading include: bifenthrin (11.2 percent), clothianidin (7.6 percent), cypermethrin (6.0 percent), fipronil (5.5 percent), cyfluthrin (3.8 percent), permethrin (2.7 percent, not shown in Fig 6), thiamethoxam (2.5 percent), spinosad (1.7 percent, not shown in Fig 6), and clothianidin from thiamethoxam (1.5 percent). The remaining “other” insecticide active ingredients combined comprise 11.6 percent of the total acute contact toxicity loading over the 23-year period.

The AITL_C_ for imidacloprid from 1995 to 2014 appears to demonstrate three defined time periods where there is stepwise increase in relative AITL_C_ contribution. Prior to 1995, imidacloprid does not contribute relevant TLU to the overall total. The first phase from 1995 to 2003 indicates that imidacloprid contributed an average of 1,595 ± 344 TLU per year for an average contribution of 11.5 percent. The second phase from 2004 to 2009 indicates that imidacloprid contributed an average of 3,441 ± 765 TLU per year for an average contribution of 18.5 percent. Finally, the third phase of increased imidacloprid use (2010–2014) indicates that this active ingredient contributed an average of 10,288 ± 1,140 TLU per year for an average contribution of 32.6 percent. The other two neonicotinoids that contribute to the total AITL_C_ (summation of TLU for all active ingredients for all years) in the 23-year period are thiamethoxam and clothianidin (both as a registered active ingredient and as a degradation product). The increasing trend in use and contribution to the total AITL_C_ begins in about 2004 for both chemicals, peaking in 2014 (the last year in our analysis) at 9.1 and 2.5 percent contribution to the total, respectively. The post-2004 increases in TLU described above is consistent with the increase in use of neonicotinoids for seed treatment at that time.

On the other hand, the chlorpyrifos AITL_C_ remains relatively constant from year-to-year over the 23 year time period with an average of 3,490 ± 810 TLU. However, when computing the contribution of chlorpyrifos to the total AITL_C_ from year-to-year, there is a steady downward trend of relative contribution. The peak contribution of chlorpyrifos to the total AITL_C_ is in 1993 (42.3 percent) and the lowest relative contributions occur from 2011 to 2014 (approximately 8 percent per year), with a gradual decline over the 23 year period.

Fipronil, a pyrazole insecticide, contributed a large proportion to the overall AITL_C_ from 1998 to 2005, with an average contribution of 14.5 ± 3.0 percent over this time period. After 2005, the use and contribution of fipronil declined rapidly because its conditional registration for use on corn was cancelled in 2010 [42], so that by 2012, the contribution was minimal (less than 0.5 percent). Four pyrethroid active ingredients bifenthrin, permethrin, zeta cypermethrin, and cyfluthrin, also contribute to the overall AITL_C_, contributing 11.2, 2.7, 6.0, and 3.8 percent over the 23-year period respectively. Individually, these active ingredients show some consistency of use and toxicity loading over the time period. Permethrin shows a steady downward trend after 2001, whereas cyfluthrin and zeta cypermethrin remain somewhat consistent from year-to-year. Bifenthrin, on the other hand, shows a large jump in use and toxicity loading contribution after 2009, with average percent contributions from 1992 to 2009 of 6.3 ± 2.6 and from 2010 to 2014 of 17.0 ± 2.4. This increase is largely due to increases in use of bifenthrin on corn, cotton, and soybeans[5–7].

#### Acute oral toxicity

With respect to AITL_O_, chlorpyrifos follows a similar trend from 1992 to 2014 as seen for acute contact toxicity with a more dramatic decrease in relative contribution over this time period (Fig 7). Over the 23-year period, the AITL_O_ for chlorpyrifos averaged 676 ± 157 TLU per year with more toxicity contribution from 1992 to 2000 (841 ± 94 TLU) per year than from 2001–2014 (569 ±76 TLU) per year. However, the relative AITL_O_ shows a steady decrease from the peak contribution of 28.6 percent in 1993 to the lowest contributions of less than 0.5 percent from 2010 to 2014. After 2003, the relative contribution of chlorpyrifos to the total AITL_O_ averaged only 0.8 percent per year. For all 23 years combined, chlorpyrifos contributed 1.4 percent (15,545 TLU) to the overall AITL_O_.

The trend in AITL_O_ from 1992 to 2014 for the neonicotinoids (Fig 7) is more complicated than seen for the AITL_C_ (Fig 6). The contribution of imidacloprid begins in 1994 and continues through 2014, loading 502,699 TLU (46.0 percent of the total TLU loading for all insecticides) into the ecosystem over this time period. In the 21-year period of imidacloprid use, there is a steady and marked increase in the *absolute* contribution of this active ingredient from year-to-year. In 1994, the AITL_O_ was 750 TLU, by 2003 it was 10,124 TLU and in 2014 it was 69,831 TLU. The *relative* contribution of imidacloprid to the total annual AITL_O_ over the same 21-year time period shows more variation. From 1995 through 2004, the average relative contribution of imidacloprid to AITL_O_ was 64.1 ±7.8 percent followed by a decrease in relative contribution from 2005 to 2014 to 43 ± 6 percent.

The decline in the relative contribution of imidacloprid after 2004 is the result of the introduction of two other neonicotinoids, thiamethoxam and clothianidin, after 2000 and 2003, respectively. As the use of these two neonicotinoids increased, the *relative* contribution of imidacloprid to the total AITL_O_ decreased. However, it is important to understand that the *absolute* contribution of the sum of these active ingredients has actually increased dramatically over this time period, and the trend suggests that this increase in acute toxicity loading on US agricultural land and surrounding areas will continue after 2014 as more acres of cropland and additional crops are treated with these insecticides. The absolute AITL_O_ of thiamethoxam increased from 315 TLU in 2002, to 3,882 TLU in 2008, to 7,700 TLU in 2014. The absolute toxicity loading of clothianidin is more pronounced, with a steady and sharp annual increase observed from 2004 to 2014. Clothianidin as an active ingredient contributed 8,928 TLU in 2004, 23,352 in 2008, and 80,083 TLU in 2014. Total clothianidin toxicity loading (active ingredient plus degradation product) is 10,632 TLU in 2004, 28,949 TLU in 2008, and 91,185 TLU in 2014. The absolute contribution of thiamethoxam and clothianidin (total) to the total AITL_O_ of all insecticides from 1992 to 2014 was 500,527 TLU or 45.8 percent of the total.

The three neonicotinoid insecticide active ingredients combined accounted for 1,003,226 TLU from 1994 to 2014, and for the entire 23-year period, contributed 91.8 percent of the total AITL_O_ of all insecticides in the US. By contrast, fipronil, which is the next most widely used insecticide active ingredient from 1992 to 2014, contributed 3.1 percent. As noted earlier, chlorpyrifos, which is the fifth most widely used insecticide active ingredient, contributed only 1.4 percent of the total AITL_O_ in the US over the 23-year period.

### AITL calculations on the basis of agricultural crops

**Fig 8:**
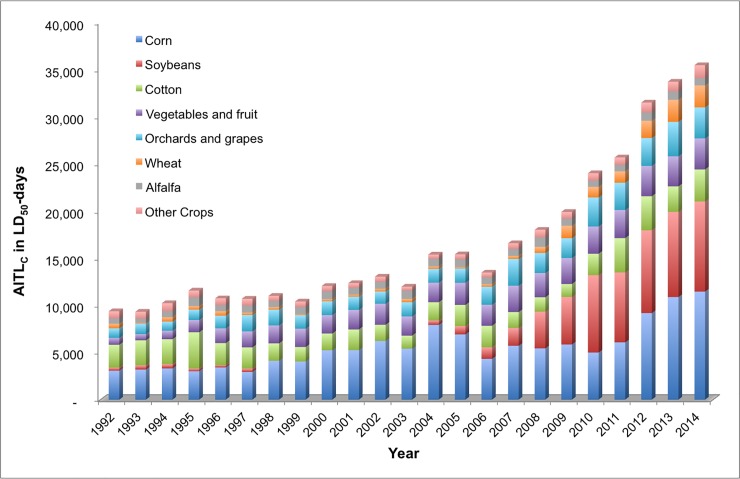
Contact acute insecticide toxicity loading (AITL_C_) by crop, 1992–2014.

**Fig 9:**
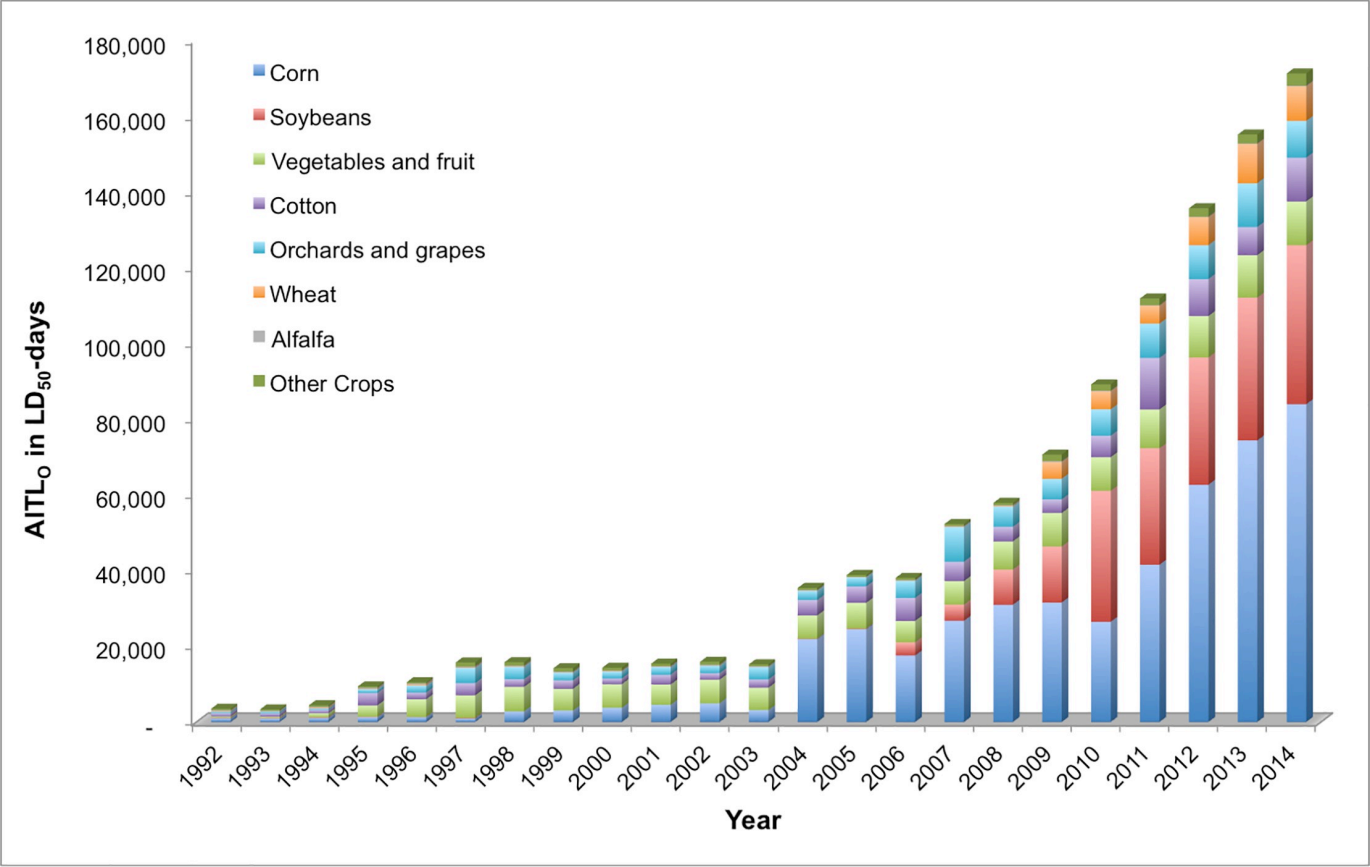
Oral acute insecticide toxicity loading (AITL_O_) by crop, 1992–2014.

The primary crops responsible for the preponderance of AITL_C_ summed over the 23-year period are corn (33.3 percent) and soybeans (15.2 percent), followed by cotton (13.9 percent), vegetables and fruit (12.9 percent), orchards and grapes (11.4 percent), alfalfa (4.5 percent), and wheat (4.0 percent) (Fig 8). Comparably, for AITL_O_, the primary crops of importance are corn (43 percent) and soybeans (19.3 percent), followed by vegetables and fruit (13.3 percent), cotton (9.0 percent), orchards and grapes (9.0 percent), and wheat (3.9 percent) (Fig 9). Collectively, crops other than those listed above (“other” crops) comprise 4.7 and 2.6 percent of the AITL_C_ and AITL_O_, respectively. Although there is some consistency in the relative contributions of the crops to the acute contact and oral toxicity loading, the absolute toxicity loading is much greater for oral acute toxicity. Overall, the total AITL_O_ for crops is 1,094,226 TLU whereas for AITL_C_ the total is 383,456 TLU, or approximately one-third of the AITL_O_, which is likely due to the greater toxicity of these insecticides via the oral route.

The USGS data set includes pesticides used as foliar sprays, seed treatments, and soil applications, but does not provide a breakdown of pounds used via different application methods. For corn, soy, and cotton, seed treatments are a primary route of application and comprise the largest contribution to increasing use [33]. Foliar uses are increasing. In 2014, there were 33 registered pesticide products containing imidacloprid for use on corn in the US; four of them approved for foliar uses [32]. For soybeans, there were 85 currently registered imidacloprid products with 54 approved for foliar uses. For cotton, there were 93 currently registered imidacloprid products with 63 approved for foliar uses.

According to the USGS, between 1998 and 2003, the survey methods used to collect the raw data for pesticide use on crops did not include a field for collecting data on insecticide application to soybean crops. Therefore, the data between 1998 and 2003 for soybeans are for herbicide application only. In 2004, USGS resumed surveying insecticide use on soybeans because it became a higher priority. The impact of this data gap is not known, but it likely would lead to a quantifiable underestimate of the relative total toxicity loading of insecticide use on soybean crops from 1992 to 2014. Interpolating the missing data is beyond the scope of our work.

## Discussion and conclusions

### Potential impacts of insecticide loading

A decline of pollinating insects is occurring worldwide [43], with negative effects for pollination of many domestic crops [44]. Several interacting factors appear to be involved, including declines in natural and diverse habitat and food supplies resulting from agricultural land use intensification, the prevalence of parasites and pathogens, exposure to chemical pesticides used predominantly in agriculture, and environmental impacts due to changes in climate [43, 45]. The impact of pesticides, in particular the neonicotinoids, on pollinator declines has received the most attention recently. For example, researchers in Great Britain used a comparable methodology to ours to show that potential honey bee deaths (the total number of LD_50_ doses applied to arable farmland) has increased six-fold to approximately 3 x 10^16^ bees over the past two decades in that country [27]. The authors attributed this result to the increasing use of neonicotinoids from 1994 to 2016. Likewise, despite its simplicity, the AITL analysis presented in this paper provides additional information in support of the hypothesis that the use of neonicotinoids on agricultural land and surrounding areas may play a primary role in the decline of insects in the US.

The AITL calculations might also be used as an analytic tool to predict future impacts of newly registered pesticide products by inputting anticipated pesticide use and toxicity to insects, then accounting for the half-life to estimate the relative potential increase in toxic loading to beneficial insects and other non-target species in the ecosystem before the product is registered. As a predictive tool, the AITL could be helpful in identifying regrettable substitutions before products are registered. In agriculture, a regrettable substitution might occur when a new pesticide product, which is developed to replace a presumably more toxic and more risky product already on the market, actually causes greater harm to the environment and non-target species than the product it is meant to replace. The AITL analysis presented here introduces the concern that the increased use of the neonicotinoid class of insecticides, presumably to replace the organophosphorus, carbamate, and pyrethroid classes of insecticides could be a case of regrettable substitution in relation to the health of beneficial insects at least and potentially to other non-target species in the environment as well. In other words, this toxicity loading analysis indicates that the neonicotinoids are potentially more harmful to pollinators and other beneficial insects than originally predicted relative to the insecticides it they are presumably replacing.

We have shown that the introduction and increasing use of the neonicotinoids from 1992 to 2014 is the primary reason for the dramatic increase in toxicity loading, in relation to pollinators and other beneficial insects and non-target arthropod species on US agricultural lands and surrounding areas. This is the result of a combination of increased use, relative toxicity, and greater persistence of neonicotinoids compared to chemical active ingredients used two decades ago. For example, in the US, imidacloprid is registered for use to control sucking insects, some chewing insects including termites, soil insects, and fleas on pets [46]. It may be applied to structures, crops, soil, and as a seed treatment as well as a topical treatment for animals. In 2016, there were 134 approved residue tolerances for imidacloprid, including crops and other applications [47]. In the 21-year period of imidacloprid use (starting in 1994), there is a steady and marked increase in the absolute contribution of this active ingredient from year-to-year reflecting its increased use over this time span (see Results). In 1994, the AITL_O_ was 750 TLU, by 2003 it was 10,124 TLU and in 2014 it was 69,831 TLU. If the use of neonicotinoids continues to increase as the use of other chemical insecticides decreases, then the absolute acute toxicity loading of imidacloprid would likely also continue to increase beyond 2014, particularly if there is approval of new crop uses of these insecticides.

Although acute insecticide toxicity loading from topical expoures (AITL_C_) presents a potential threat to beneficial insects and other nontarget species, the acute insect toxicity loading from oral exposures (AITL_O_) might present an even greater potential threat. This is due to the higher level of toxicity (i.e., lower LD_50_s), increased persistence (i.e., longer half-lives), and the potential for greater relative exposure via the oral route. In absolute terms, over the time period from 1992 to 2014, the total AITL_C_ is 383,456 TLU, whereas the total AITL_O_ is 1,094,226 TLU, which is nearly three times greater than the AITL_C._ We found that three neonicotinoid insecticide active ingredients (imidacloprid, thiamethoxam, and clothianidin) combine to contribute 91.8 percent of the total AITL_O_ of all insecticides in the US. As noted earlier, chlorpyrifos, which is the fifth most widely used insecticide active ingredient, contributed only 1.4 percent of the total AITL_O_ in the US from 1992–2014.

### Limitations of the AITL method

Pesticide use by pounds (kilograms) applied or acres treated does not provide a comprehensive estimate of toxicity loading to an ecosystem. Factors such as persistence, toxicity, application methods and timing, exposure routes, and mechanisms of dissipation from the application site all influence the net toxicity experienced by insects in the ecosystem. The data needed to do an analysis that incorporates all of these factors is largely unavailable.

As noted previously, the AITL analysis does not account for trends in pesticide application in seed treatments nor does it quantify the actual or estimated exposure dose of an insecticide after seed treatment. In a risk-based approach, omitting these factors may result in an overestimation of hazard potential to pollinators and other non-target species from exposure to insecticides applied as seed treatments. Therefore, a more refined approach would be required to estimate actual hazard impacts from seed treatments, in particular for the use of neonicotinoids. This level of refinement is difficult but it would provide a more accurate assessment of the impact of these insecticides on US agricultural land and surrounding areas. Furthermore, other factors that contribute to toxicity loading, such as the application method and the change in the types of application methods used over time, were not evaluated in this analysis. Different pesticide application methods (e.g., spray, soil drench, granules, coated seeds) result in different exposure potential for aquatic versus terrestrial ecosystems, which is not assessed in this analysis. Because our analysis does not account for the timing of insecticide application, the AITL does not identify “peaks” and “ebbs” in toxicity over time relative to the exact time and mode of application. Instead, our analysis assumes a steady state from one application to another. This simplifying assumption does not affect comparisons of insecticide toxicity loading from year-to-year but it does diminish the method’s ability to identify specific time periods when toxicity loading might be the most damaging to the ecosystem in US agricultural land and surrounding areas.

As is, on the one hand the AITL analysis likely overestimates acute toxicity hazard to pollinators and other beneficial insects because of the simplifying assumptions used. On the other hand, the AITL analysis likely underestimates *actual* toxicity hazard because it does not account for sublethal effects, movement of pesticides offsite, or potential synergistic impacts of pesticides used in combination in the field. Nevertheless, as a screening tool, the results of an AITL can assist regulators in identifying chemicals of concern for further evaluation.

### Other toxicity concerns

#### Sublethal toxicity

We were limited to using acute lethal toxicity (LD_50_) as an endpoint in our AITL analysis because sublethal toxicity studies of pesticides in honey bees are currently not required for registration in the US, although US EPA has published guidance [48]. Therefore, the LD_50_ dataset on honey bees is the only insect toxicity data available for a large number of pesticides registered for use in the US, which allowed us to compare historical trends for all relevant insecticide classes. Lethality is at the extreme end of the toxicity spectrum and using mortality as the endpoint for the AITL analysis or for risk assessment is a blunt instrument for evaluating the impact of pesticides on the ecosystem. Because actual toxicity risks to pollinators and other non-target species would be higher using sublethal toxicity doses, the impacts of pesticides on beneficial insect populations and other non-target species is underestimated when limited to using lethal doses.

The AITL analysis can be modified for the input of sublethal toxicity doses when data exist. For some of the neonicotinoids, the dataset for sublethal effects is adequate to allow for a comparison of the toxic effects of these insecticides at high dose levels to the more sensitive sublethal effects at lower doses. However, the availability of a robust database for pesticide active ingredients is the exception, not the rule. Very few studies are available regarding the sublethal effects of organophosphorus, carbamate, and organochlorine pesticides on insects, so it is difficult to do a comparative analysis with these chemicals.

In the case of honey bees, reported sublethal effects from neonicotinoid exposure in laboratory and field studies include impaired reproduction, altered immune function, inability to navigate effectively, and behavioral changes in essential colony activities leading to decreased colony health and survival [4]. We present a listing of sublethal toxicity values (in units of ng/bee) taken from representative laboratory and field studies in the published literature of neonicotinoids in honey bees in the supporting materials that accompany this paper (S2 Appendix). The dose levels reported for lethality (LD_50_) when compared to the lowest observed effect concentration (LOEC) for sublethal toxicity are noticeably higher (Table 3). For imidacloprid, the most heavily used of this class of insecticides, the lethal dose for the oral route is 37 times the sublethal LOEC and for contact toxicity the lethal dose is 320 times the sublethal LOEC. In addition, imidacloprid is also one of the more environmentally persistent pesticide active ingredients used today, enhancing the potential for sublethal exposures.

**Table 3:** Comparison of honey bee LD50’s with sublethal lowest observed effect concentrations (LOEC) for neonicotinoids and related compounds.

Active Ingredient	Field/Soil Half-life (days)	LD50 Contact (μg/bee)	LD50 Oral (μg/bee)	LOEC Contact (μg/bee)	LOEC Oral (μg/bee)
Acetamiprid	3	8.1	15	0.1*	0.1*
Clothianidin	121	0.044	0.0079	0.0022*	0.0005–0.0009
Dinotefuran	75	0.03	0.04	0.0075*	NA
Imidacloprid	174	0.032	0.0037	0.0001*	0.0001–0.0015
Sulfoxaflor	2.2	0.38	0.15	NA	NA
Thiacloprid	18	26	18	NA	0.0013*
Thiamethoxam	39	0.02	0.005	0.0001–0.004	0.0004–0.002

Half-life and LD_50_ data transferred from S1 Appendix, and LOEC data from S2 Appendix.

* No range available.

NA Not available

The results of an insecticide toxicity loading analysis of sublethal toxicity would likely demonstrate that the absolute sublethal TLU for the neonicotinoids would be noticeably higher than the AITL, indicating a greater overall toxicity loading on agricultural land and surrounding areas when compared to the acute TLU calculated from using the LD_50_ values. The relative toxicity loading contribution of the different neonicotinoids might also change, although it would be difficult to predict the outcome without doing the calculations. When sublethal toxicity data exist, the calculation of the toxicity loading using these values rather than LD_50_s would be informative.

#### Pesticide movement offsite

One of the limitations of our AITL analysis is that, while we account for total pesticides applied to agricultural land using pesticide use estimates published by USGS (see Methods), we cannot quantify insecticide toxicity loading in the impact zone beyond the boundaries of agricultural land or indeed the greater likelihood of in-field exposure to highly systemic and persistent insecticides such as neonicotinoids. This omission will tend to underestimate the toxicity loading of pesticides on land surrounding agricultural fields and in surface water and other waterways distal to the fields. Including persistence in this evaluation of overall toxicity is important because persistent pesticides have a greater potential and tendency to move offsite unchanged into surrounding fields, land, surface water, and other waterways outside of the agricultural fields where they are directly applied [12, 49].

Insects in an ecosystem can be exposed to insecticides through a number of different routes. Pesticides run off into surface waters with precipitation or irrigation, leach into groundwater, and drift as dusts or on soil particulates from the application site in the air, affecting insects both on and near the application site. Direct insecticide application exposes insects on the crop being treated as well as those in near-field vegetation [50] waterways [16, 17], and pooled surface water [51]. Soil-borne larvae or adult insects can be exposed through direct sprays, granular or soil-drench applications, or through migration of residues remaining in the soil from the planting of coated seeds. Insects consuming pollen or nectar, or sucking or chewing insects consuming plants both on and near the application site are exposed through oral consumption. Aquatic species living a substantial distance from the application site can be exposed through drift and runoff from treated fields that contaminates waterways [52].

While the toxic effects of an insecticide are highest at the application site where the concentrations are highest, dissipation pathways such as irrigation or rainwater runoff to surface waters can carry toxicologically significant amounts of pesticides into waterways. For example, surface water contamination has been shown to negatively impact beneficial insects and other non-target species [14, 53]. Because the neonicotinoid insecticides are highly water soluble and persistent, their potential for off-site impacts on aquatic organisms is high.

Pesticides in airborne field dust, which is generated during and shortly after application to agricultural fields, also presents a potentially important source of exposure to beneficial insects. In particular, neonicotinoid-treated seeds (e.g., soybean) contain high concentrations of neonicotinoids, which when mixed with field dust, can move offsite in the air depositing on surrounding land, flowers, and other vegetation potentially exposing pollinators and other non-target insects [52, 54, 55]. Additionally, soils in fields treated with long half-life insecticides year after year may increase in toxicity over time, as the insecticide accumulates in the soil [56].

#### Synergistic effects

This analysis is also limited by the fact that virtually all environmental toxicology data on pesticide active ingredients are for a single chemical only and not for a combination of chemicals. However, pesticide products applied to agricultural fields in the US are frequently used in combination with other products and chemicals with the potential for concurrent and/or sequential exposure to more than one chemical on a regular basis. Furthermore, environmental exposures to chemicals occur via a variety of pathways (e.g., contact with wildflowers and other vegetation, water, soil, air, and bioaccumulation in the food chain), often with multiple exposure routes (e.g., oral and contact). Generally, beneficial insects such as honey bees are exposed to combinations of pesticide products when they contact pollen and nectar and other vegetation in the fields [57, 58].

Combinations of active ingredients and other chemicals (the so-called “inerts”) in pesticide products have been measured in honey bees, hive wax, wildflowers, and pollen in the US and Europe [59–63]. From these and other studies it has been shown that mixtures of neonicotinoids in combination with a broad range of other pesticide active ingredients and other chemicals have been reported in bees, beehive matrices (pollen, nectar, honey, wax), and food sources, in some cases with as many as 121 to 150 different chemicals. There is also growing evidence that mixtures of chemicals such as insecticides, interactions of bee pathogens and parasites, and combinations of these stressors can interact together in additive or in a synergistic manner to increase morbidity and mortality in bees [63–74].

Generally, the outcome of mixing chemicals and/or biological agents together is nearly impossible to predict with the limitations in capability and throughput of the currently available toxicity testing methods. The lack of information and knowledge about the behavior and toxicity of chemical mixtures in biological systems is important to acknowledge, as is the specific impact of these synergistic relationships to overall bee and colony health as well as other beneficial insects and non-target species. The AITL assessment presented here is based on the chemical and toxicological properties of individual chemical active ingredients and does not account for chemical mixtures. Therefore, any interactions of chemicals in a mixture, beyond perhaps simple additivity, would be underrepresented in our estimates.

### Conclusions

Based on our analysis of the Acute Insecticide Toxicity Loading (AITL) of pesticides applied to US agricultural lands and surrounding areas from 1992 through 2014, using honey bees as an indicator species to assess toxicity to a wide range of terrestrial insects, we conclude:

1. The toxicity loading of insecticides on agricultural land and surrounding areas has increased by approximately 50-fold over the last two decades producing both direct and indirect effects on associated ecosystems. Although current-use pesticides are applied at lower application rates per acre, they are more toxic to insects and persist in the environment for up to several weeks or longer, thus creating a persistent toxicity load in plants, soils, and surface waters that is substantially higher than that experienced by insects 20 or more years ago.
2. The neonicotinoid insecticides, in particular imidacloprid, clothianidin, and thiamethoxam, are primarily responsible for this increased toxicity loading, accounting for 61percent (via contact toxicity) to 99 percent (via oral toxicity) of the total toxicity loading of all insecticides in 2014. Oral exposures appear to be of greater concern because of the relatively higher toxicity (i.e. low LD_50_s) and greater likelihood for exposure from residues in pollen, nectar, guttation water, and other environmental media. However, because the AITL does not incorporate quantified exposures, a statistical comparison of toxicity loading via different exposures routes is beyond the scope of this paper.
3. The crops most responsible for the increase in AITL are corn and soybeans, with particularly large increases in relative soybean contributions to AITL between 2010 and 2014.
4. The total oral AITL of all insecticides applied over the 23-year period is an order of magnitude greater than the total contact AITL.
5. This increase in toxicity loading is consistent with the reduction in beneficial insect and insectivorous bird populations observed in recent years. However, a more refined analysis of risk, including quantified exposures and factoring of application methods would be required to demonstrate a clear association.
6. The introduction and increased use of the neonicotinoids in the late 1990s appears to be an example, in hindsight, of a regrettable substitution that might have been avoided had proper predictive analytical tools been available and applied prior to the approval of the registration of these pesticide products.
7. FIFRA mandates that an applicant for the registration (licensing) of a new pesticide product must show that the use of a pesticide as specified “will not generally cause unreasonable adverse effects on the environment” [75]. Based on our screening level analysis of toxicity loading of insecticides on US agricultural land and surrounding areas, it is our scientific opinion that existing regulations for the registration of new pesticide active ingredients in the US are not yet adequate to effectively prevent the introduction of new chemicals that are detrimental to beneficial insect species such as the pollinators and other non-target species.
8. Using methodology such as the AITL screening analysis early in the registration process of new active ingredients or in approving new agricultural uses would provide useful metrics with which to predict catastrophic harm to the environment resulting from the application of chemical pesticides on agricultural land. Expansion of the testing requirement to include sublethal toxicity testing in honey bees (or other surrogate arthropods) would provide a more refined estimate of the true risk of the introduction of new pesticide chemicals. Furthermore, implementation of a comprehensive surveillance and use reporting system for pesticides that have the potential to disrupt the ecosystems on agricultural lands and surrounding areas, including pesticide use as seed coatings would additionally enhance regulators’ abilities to assess and prevent potential adverse effects before ecosystems are damaged.

## Supporting information

S1 Appendix Environmental half-lives and LD50 values used in AITL assessment. (PDF)

S2 Appendix Representative lowest observed effect concentrations (LOEC) for neonicotinoid sublethal toxicity in honey bees. (PDF)
