# Supplementary material for: An assessment of acute insecticide toxicity loading (AITL) of chemical pesticides used on agricultural land in the United States
Source: PLoS One. 2019 Aug 6;14(8):e0220029. doi: 10.1371/journal.pone.0220029 (PMC6684040; doi:10.1371/journal.pone.0220029)
Supplement: S1 Appendix — (PDF) [file pone.0220029.s001.pdf]

S1 Appendix. Environmental half-lives and LD_{50} values used in AITL assessment

Active ingredient	Half-life (days)	Study Type	Citation	Contact LD50 (µg/bee)	Oral LD50 (µg/bee)	Citation
Abamectin	1	EU Field	[1]	0.0022	0.0063	[3] [4] [5]
Acephate	3	EU Field	[1]	1.1	1.4	[1] [5] [6]
Acetamiprid	3	EU Field	[1]	8.1	14.8	[1] [3] [5] [8]
Aldicarb	2	EU Field	[1]	0.31	0.12	[1] [5] [6] [7] [9]
Aldrin	365	EU Field	[1]	0.35	0.35	[1] [9] [16]
Azadirachtin	26	DPR Aerobic	[2]	25	8.1	[4] [5]
Azinphos-methyl	10	EU Soil	[1]	0.12	0.15	[1] [5] [15] [24]
Bifenazate	4.7	EU Field	[1]	7.9	110.0	[3] [5]
Bifenthrin	86.8	EU Field	[1]	0.074	0.29	[1] [3] [5] [6]
Carbaryl	16	EU Soil	[1]	0.54	0.18	[1] [5] [6] [7] [15] [32]
Carbofuran	14	EU Field	[1]	0.12	0.05	[1] [5] [6] [9]
Chlorantraniliprole	210	EU Soil	[1]	100	104	[1] [3] [5]
Chlordane	365	EU Soil	[1]	6.9	NR	[1] [5] [9] [15]
Chlorethoxyphos	3	EU Field	[1]	0.07	NR	[1] [5]
Chlorpyrifos	21	EU Field	[1]	0.059	0.25	[1] [3] [5] [6] [7] [15]
Clothianidin	121.2	EU Field	[1]	0.041	0.0079	[1] [3] [5] [6] [10]
Cyfluthrin-beta	13	EU Field	[1]	0.013	0.04	[1] [5] [6] [20]
Cyhalothrin-lambda	25	EU Soil	[1]	0.06	0.26	[1] [6] [7] [16] [23]
Cypermethrin	35	EU Field	[1]	0.027	0.058	[1] [3] [5] [6] [7] [18] [19]

Active ingredient	Half-life (days)	Study Type	Citation	Contact LD50 (μg/bee)	Oral LD50 (μg/bee)	Citation
Cypermethrin, zeta	10	EU Field	[1]	0.027	0.058	[1] [3] [5] [6] [7] [18] [19]
DDT	6200	EU Soil	[1]	5.5	5.3	[5] [6] [15]
Deltamethrin	21	EU Field	[1]	0.038	0.011	[1] [3] [5] [6] [7][16] [23]
Demeton	2.7	DPR Aerobic	[2]	2.6	NR	[1] [5]
Diazinon	18.4	EU Field	[1]	0.28	0.17	[1] [5] [6] [15]
Dicofol	80	EU Soil	[1]	12	50	[1] [5] [15]
Dicrotophos	28	EU Soil	[1]	0.076	0.068	[1] [5] [15]
Dienochlor	300	EU Soil	[1]	36	NR	[1]
Dimethoate	7.2	EU Field	[1]	0.12	0.12	[1] [5] [6] [7] [15]
Dinotefuran	75	EU Field	[1]	0.03	0.04	[1] [5] [6] [13]
Disulfoton	30	EU Soil	[1]	4.5	28	[1] [5] [7] [15]
Emamectin benzoate	211	DPR Aerobic	[2]	0.0035	NR	[3][5]
Endosulfan	86	EU Field	[1]	6.7	6.5	[5] [6] [7] [15]
Esfenvalerate	44	EU Field	[1]	0.04	0.21	[1] [3] [5] [6] [21]
Ethion	90	EU Soil	[1]	20.60	NR	[1] [5]
Ethoprop	23	EU Field	[1]	4.07	NR	[1] [3] [5]
Fenamiphos	1.8	EU Field	[1]	1.08	0.45	[1] [5]
Fenbutatin-oxide	95	EU Field	[1]	3982	200	[1] [5]
Fenoxycarb	5.94	EU Field	[1]	204	1000	[1] [3] [5]
Fenpropathrin	28	EU Field	[1]	0.050	NR	[1]
Fenvalerate	40	EU Soil	[1]	0.32	NR	[1] [5] [16]
Fipronil	65	EU Field	[1]	0.009	0.003	[1] [5] [6] [7]
Fonofos	40	EU Field	[1]	6.0	8.4	[1] [5] [15]

Active ingredient	Half-life (days)	Study Type	Citation	Contact LD50 (µg/bee)	Oral LD50 (µg/bee)	Citation
Hydramethylnon	7	EU Field	[1]	67	NR	[1] [5]
Imidacloprid	174	EU Field	[1]	0.043	0.0037	[1] [3] [5] [6] [7] [11]
Indoxacarb	20	EU Field	[1]	0.10	0.18	[1] [3] [5]
Isofenphos	150	EU Soil	[1]	0.049	NR	[5]
Lindane	148	EU Field	[1]	0.36	0.12	[1] [5] [6] [15]
Malathion	1	EU Field	[1]	0.21	0.38	[1] [3] [5] [15]
Methamidophos	4	EU Field	[1]	1.37	0.22	[1] [9]
Methidathion	7	EU Field	[1]	0.237	NR	[1] [5] [9] [11]
Methiocarb	35	EU Field	[1]	0.30	0.47	[1] [3] [5] [9]
Methomyl	7	EU Soil	[1]	0.47	0.72	[1] [3] [5] [6] [9]
Methoxychlor	120	EU Soil	[1]	23.6	5.0	[1] [5]
Methyl parathion	10	EU Field	[1]	0.22	NR	[5] [9] [16]
Mevinphos	1.2	EU Soil	[1]	0.07	0.027	[5] [15]
Monocrotophos	30	EU Field	[1]	0.36	0.02	[1] [9]
Naled	1	EU Soil	[1]	0.48	NR	[5] [9]
Oxamyl	11	EU Field	[1]	1.32	0.32	[1] [5] [7] [9] [15]
Oxydemeton-methyl	5	EU Field	[1]	0.90	0.25	[1] [5] [9] [15]
Parathion	17	EU Field	[1]	0.11	NR	[5] [9] [23]
Permethrin	42	EU Field	[1]	0.075	0.19	[1] [5] [6] [7] [9] [15]
Phorate	63	EU Field	[1]	0.89	0.44	[1] [5] [6] [9] [15]
Phosmet	7	EU Field	[1]	0.79	0.37	[1] [3] [5] [6] [7] [9]
Phosphamidon	12	EU Field	[1]	1.46	NR	[1] [5] [7] [9]
Pirimicarb	9	EU Field	[1]	35.5	3.7	[1] [3] [9] [10] [15]

Active ingredient	Half-life (days)	Study Type	Citation	Contact LD50 (µg/bee)	Oral LD50 (µg/bee)	Citation
Profenofos	7	EU Field	[1]	0.095	NR	[1] [5]
Propargite	16.7	EU Field	[1]	38	100	[1] [5]
Pyrethrins	8	DPR Aerobic	[2]	0.094	0.15	[3] [5] [15]
Pyridaben	29	EU Field	[1]	0.13	1.58	[1] [5] [6]
Spinetoram (mixture of J and L)	16.1	EU Soil	[1]	0.026	0.082	[1] [3] [5]
Spinosad (mixture ofA and D)	14	DPR Aerobic	[2]	0.0038	0.055	[4] [5] [7] [17]
Spirodiclofen	7	EU Soil	[1]	200	196	[1] [3] [5]
Spiromesifen	2.1	EU Field	[1]	200	792	[1] [5]
Sulfoxaflor	2.2	EU Soil	[1]	0.23	0.16	[1] [5] [14] [22]
Tau-fluvalinate	4	EU Soil	[1]	10	12.6	[1] [3] [7]
Tefluthrin	27.1	EU Field	[1]	0.22	1.81	[1] [3] [5]
Terbufos	12	EU Field	[1]	0.41	4.09	[1] [5] [9]
Thiacloprid	18	EU Field	[1]	26	18	[1] [3] [5] [7]
Thiamethoxam	39	EU Field	[1]	0.015	0.005	[1] [3] [5] [6] [7]
Toxaphene	9	EU Field	[1]	50	20	[5]
Tralomethrin	3	EU Soil	[1]	0.13	NR	[1] [5]
Trichlorfon	18	EU Soil	[1]	4.40	0.4	[1] [5] [7] [25]

NR: No values identified or reported in the published literature.

References

[1] University of Hertfordshire. The Pesticide Properties Data Base (PPDB). Developed by the Agriculture and Environment Research Unit, University of Hertfordshire. Version 2017; Nov 30. Available from: http://sitem.herts.ac.uk/aeru/ppdb/en/index.htm.

[2] Bergin R. 2015 Status report Pesticide Contamination Prevention Act, annual report. CA Department of Pesticide Regulation Report PCPA15. 2016. Available from: http://www.cdpr.ca.gov/docs/emon/pubs/ehapreps/report_pcpa15.pdf.

[3] National Institute for Agricultural Research (France). Database on plant protection substances. Agence nationale de sécurité sanitaire de l’alimentation, de l’environnement et du travail 2017;Jun 6. Available from: http://www.agritox.anses.fr/index2.php. http://www.agritox.anses.fr/index2.php.

[4] BPDB: Bio-Pesticides "FOOTPRINT" database. University of hertfordshire. Available from: https://sitem.herts.ac.uk/aeru/bpdb/index.htm.
[5] US EPA EcoTox. OPP pesticide ecotoxicty database. US Environmental Protection Agency, Regional IPM Centers. 2017. Available from: http://www.ipmcenters.org/ecotox/DataAccess.cfm.
[6] Sanchez-Bayo F, Goka K. (2014). Pesticide Residues and Bees – A Risk Assessment. PLoS ONE 9(4): e94482. doi:10.1371/journal.pone.0094482.
[7] Arena M, Sgolastra F. (2014). A meta-analysis comparing the sensitivity of bees to pesticides: Database on the LD50 acute and oral and LC50 for honeybees, bumblebees, solitary bees and stingless bees. Ecotoxicology 2014; Electronic supplementary material DOI 10.1007/s10646-014-1190-1. Available from: https://link.springer.com/article/10.1007%2Fs10646-014-1190-1.
[8] US EPA 2005. Acetamiprid new use (cucurbits, stone fruits and tree nuts): Environmental fate and effects risk assessment. US Environmental Protection Agency, Office of Prevention, Pesticides and Toxic Substances, July 27, 2004. Available from: https://www3.epa.gov/pesticides/chem_search/cleared_reviews/csr_PC-099050_27-Jul-05_a.pdf.
[9] Atkins EL, Kellum D, Atkins KW (1981) Reducing pesticide hazards to honey bees: Mortality prediction and integrated management strategies. Univ Calif Div Agric Sci Leafl 2883.
[10] US EPA 2003. Pesticide fact sheet: clothianidin. US Environmental Protection Agency, Office of Prevention, Pesticides and Toxic Substances. May 30, 2003. Available from: https://www3.epa.gov/pesticides/chem_search/reg_actions/registration/fs_PC-044309_30-May-03.pdf.
[11] US EPA 1994. Registration for imidacloprid (NTN 33893). US Environmental Protection Agency, Office of Prevention, Pesticides and Toxic Substances. March 10, 1994. Available from: https://www3.epa.gov/pesticides/chem_search/cleared_reviews/csr_PC-129099_10-Mar-94.pdf.
[13] US EPA 2004. Fact sheet: dinotefuran. US Environmental Protection Agency, Office of Prevention, Pesticides and Toxic Substances, September 2004. Available from: https://nepis.epa.gov/Exe/ZyPURL.cgi?Dockey=P100BIDI.TXT.
[14] US EPA 2010. Environmental fate and ecological risk assessment for sulfoxaflor registration. 2010. Sulfoxaflor_EPA-HQ-OPP-2010-0889-0022.
[15] Stevenson JH. The acute toxicity of unformulated pesticides to worker honey bees (Apis mellifera L.). Plant Pathol. 1978; 27: 38–40. doi: 10.1111/j.1365-3059.1978.tb01070.x.
[16] International Programme on Chemical Safety (IPCS). INCHEM Database. Last accessed on Dec 19, 2017. Available from: http://www.inchem.org/.
[17] Mayes MA, Thompson GD, Husband B, Miles MM. Spinosad toxicity to pollinators and associated risk. In: Reviews of Environmental Contamination and Toxicology. 2003; Reviews of Environmental Contamination and Toxicology, vol 179. Springer, New York, NY. doi.org/10.1007/0-387-21731-2_2.
[18] US EPA, 2008. Revised reregistration eligibility decision (RED): Cypermethrin. US Environmental Protection Agency, Office of Prevention, Pesticides and Toxic Substances. EPA OPP-2005-0293. June 14, 2006, revised January 14, 2008.
[19] European Food Safety Authority (EFSA). Draft renewal assessment report prepared accroding to regulation (EC) number 1107/2009 for alpha-cypermethrin. Volume 1, April 2017. Available from: https://www.efsa.europa.eu/en/consultations/call/170809.

[20] European Food Safety Authority (EFSA). Renewal assessment report for beta-cyfluthrin. Volume 1, March 7, 2017. Available from: https://www.efsa.europa.eu/en/consultations/call/170407.
[21] European Food Safety Authority (EFSA). Conclusion on the peer review of the pesticide risk assessment of the active substance esfenvalerate. European Food Safety Authority (EFSA), Parma, Italy. EFSA Journal 2014;12(11):3873. Available from: https://www.efsa.europa.eu/en/efsajournal/pub/3873.
[22] European Food Safety Authority (EFSA). Conclusion on the peer review of the pesticide risk assessment of the active substance sulfoxaflor. European Food Safety Authority (EFSA), Parma, Italy. EFSA Journal 2014;12(5):3692. Available from: https://www.efsa.europa.eu/en/efsajournal/pub/3692.
[23] EXTOXNET. The Extension Toxicology Network. EXTOXNET is a cooperative effort of University of California-Davis, Oregon State University, Michigan State University, Cornell University, and the University of Idaho. Available from: http://extoxnet.orst.edu/.
[24] Danka RG, Rindere TE, Hellmich RL, Collins AM. Comparative toxicities of four topically applied insecticides to Africanized and European honey bees (Hymenoptera: Apidae). J Econ Entomol. 1986; 79: 18-21.
[25] Ahmad Z, Johansen, CA. Selective toxicity of carbophenothion and trichlophon to the honey bee and the alfalfa-cutting bee. Environ Entomol. 1973; 2:27-30.
