# Supplementary material for: An assessment of acute insecticide toxicity loading (AITL) of chemical pesticides used on agricultural land in the United States
Source: PLoS One. 2019 Aug 6;14(8):e0220029. doi: 10.1371/journal.pone.0220029 (PMC6684040; doi:10.1371/journal.pone.0220029)
Supplement: S2 Appendix — (PDF) [file pone.0220029.s002.pdf]

S2 Appendix. Representative lowest observed effect concentrations (LOEC) for neonicotinoid sublethal toxicity in honey bees.

Neonicotnoid (a.i.)	Test Doses	Mode of Exposure	Time of exposure	LOEC	Endpoint	Study Type	Reference
Acetamiprid	0, 0.1, 0.5, 1.0 μg/bee	Oral	Acute	≤100 ng/bee	Impaired long-term retention of olfactory learning	Laboratory	[1, 2]
Acetamiprid	0, 0.1, 1.0 μg/bee	Oral	Acute 24 hrs	≤100 ng/bee	Increase in responsiveness to water	Laboratory	[2]
Acetamiprid	0, 0.1, 0.5, 1.0 μg/bee	Topical	Acute	≤100 ng/bee	Increased locomotor activity and water-induced proboscis extension reflex	Laboratory	[1]
Clothianidin	0, 0.1, 0.5, 1.0, 2.0 ng/bee	Oral	Acute 30-90 min	0.5 ng/bee	Significant change in locomotor activity	Laboratory	[3]
Clothianidin	0, 0.06, 0.3, 0.6, 0.9, 3.0, 6.0, and 12.0 ng/bee	Oral	Acute	0.9 ng/bee	Changes specificity of the early long-term memory (24 h)	Laboratory	[3]
Clothianidin	0, 0.03, 0.06, 0.12, 0.24 ng/bee	Oral	Chronic up to 4 weeks	0.12 ng/bee	Increased AChE activity with associated hyperactivity and decreased survival	Laboratory and Field	[4]
Clothianidin	0, 0.05, 0.5, 1.0, 2.0 ng/bee	Oral	Acute, single dose	0.5 ng/bee	Increase the time spent at feeder, flight time to feeder or to hive, time in hive between trips and trip duration	Field	[5]
Clothianidin	0, 2.5 ng/bee	Oral	Acute	≤2.5 ng/bee	Modifications in the length and directional components of vector flight and homing flight	Field	[6]
Clothianidin	0.545-10.9 ng/bee	Topical	Acute 30 minutes	2.18 ng/bee	Reduction in successful homing flight	Field	[7]

Neonicotnoid (a.i.)	Test Doses	Mode of Exposure	Time of exposure	LOEC	Endpoint	Study Type	Reference
Dinotefuran	0, 1.9-37.5 ng/bee	Topical	Acute 30 minutes	7.5 ng/bee	Reduction in successful homing flight	Field	[7]
Imidacloprid	0, 10, 100, 1000 µM	Oral	Subchronic 6 days	1.3 ng/bee	Impairment of various aspects of olfactory learning and memory formation	Laboratory	[8]
Imidacloprid	0, 2, 10 ng/bee	Oral	Acute, a few hours	≤2 ng/bee	Reduction in mobility (moving time ratio and the speed of movements)	Laboratory	[9]
Imidacloprid	0, 0.1, 1.0 ng/bee	Oral	NR	≤0.1 ng/bee	Impairment of olfactory learning	Laboratory	[10]
Imidacloprid	0, 0.1, 1.0 ng/bee	Oral	Acute 4 hrs	≤0.1 ng/bee	Changed in the number of trials to habituation in an age-dependent manner	Laboratory	[11]
Imidacloprid	0, 0.01, 0.1, and 1.0 ng/bee	Oral	Subchronic 8-10 days	0.1 ng/bee	Increased mortality of workers	Laboratory	[12]
Imidacloprid	~0.8-81 ng/bee	Oral	Chronic	~≤1 ng/bee	Altered foraging behavior	Laboratory	[13]
Imidacloprid	0, 0.21, 2.16 ng/bee	Oral	Acute 1 - 24 hrs	≤ 0.21 ng/bee	Higher sucrose response thresholds (1 hr) and a reduction in waggle dance circuits (24 hrs)	Laboratory	[14]
Imidacloprid	0, 0.453 ng/bee	Oral	Acute 24 hrs	≤0.453 ng/bee	Disruption of the righting reflex and increased grooming	Laboratory	[15]
Imidacloprid	0, 0.08, 0.16, 0.24, 0.30 ng/bee	Oral	Chronic up to 4 weeks	≤0.08 ng/bee	Increased AChE activity with associated hyperactivity and decreased survival	Laboratory and Field	[4]
Imidacloprid	0, 0.15, 1.5, 3.0, 6.0 ng/bee	Oral	Acute, single dose	1.5 ng/bee	Increase the time spent at feeder, flight time to feeder or to hive, time in hive between trips and trip duration	Field	[5]

**Neonicotnoid (a.i.)**	**Test Doses**	**Mode of Exposure**	**Time of exposure**	**LOEC**	**Endpoint**	**Study Type**	**Reference**
Imidacloprid	0, 7.5, 11.25 ng/bee	Oral	Acute	≤7.5 ng/bee	Modifications in the length and directional components of vector flight and homing flight	Field	[6]
Imidacloprid	0, 1.25, 2.5, 5, 10, and 20 ng/bee	Topical	Acute	≤1.25 µg/bee	increases the motor activity and facilitates the PER habituation independently of time.	Laboratory	[16]
Thiacloprid	0, 1.25 µg/bee	Oral	Acute	≤1.25 µg/bee	Modifications in the length and directional components of vector flight and homing flight	Field	[6]
Thiamethoxam	0, 1.3 ng/bee	Oral	NR	≤1.3 ng/bee	Locomotor deficit	Laboratory	[17]
Thiamethoxam	0, 0.428, 4.28 ng/bee	Oral	Subchronic 8 days	≤0.428 ng/bee	Condensed cells in mushroom bodies and optical lobes, cytoplasm vacuolization, increased apocrine secretion, increased cell elimination	Laboratory	[18]
Thiamethoxam	0, 1.34 ng/bee	Oral	Acute	≤1.34 ng/bee	Reduction in successful homing flight and increased mortality for foragers	Field	[19]
Thiamethoxam	0, 1.95, 2.90, 3.71, and 4.53 ng/bee	Oral	Acute (1-48 hrs)	≤1.96 ng/bee	Decreased flight duration, distance, and average velocity	Field	[20]
Thiamethoxam	0, 3.8 ng/bee	Topical	NR	≤3.8 ng/bee	Locomotor deficit	Laboratory	[17]
Thiamethoxam	0, 0.1, 1.0 ng/bee	Topical	Acute 24 hrs	≤0.1 ng/bee	Significant decrease of olfactory memory	Laboratory	[2]
Thiamethoxam	0, 2.56, 5.12, 51.2 ng/bee	Topical	Acute 48 hours	≤2.56 ng/bee	Changes in biochemical markers for toxicity	Laboratory	[21]

References

1. El Hassani A, Dacher M, Vincent G, Lambin M, Gauthier M, Armengaud C. Effects of sublethal doses of acetamiprid and thiamethoxam on the behavior of the honeybee (Apis mellifera). Arch Environ Contam Toxicol. 2008;54:653–61. doi: 10.1007/s00244-007-9071-8.
2. Aliouane Y, El Hassani AK, Gary V, Armengaud C, Lambin M, Gauthier M. Subchronic exposure of honeybees to sublethal doses of pesticides: effects on behavior. Environ Toxicol Chem. 2009;28(1):113-22. doi: 10.1897/08-110.1.
3. Alkassab AT, Kirchner WH. Assessment of acute sublethal effects of clothianidin on motor function of honeybee workers using video-tracking analysis. Ecotoxicol Environ Saf. 2018;147:200-5. doi: 10.1016/j.ecoenv.2017.08.047.
4. Boily M, Sarrasin B, Deblois C, Aras P, Chagnon M. Acetylcholinesterase in honey bees (Apis mellifera) exposed to neonicotinoids, atrazine and glyphosate: laboratory and field experiments. Environ Sci Pollut Res Int. 2013;20(8):5603-14. doi: 10.1007/s11356-013-1568-2.
5. Schneider CW, Tautz J, Grunewald B, Fuchs S. RFID tracking of sublethal effects of two neonicotinoid insecticides on the foraging behavior of Apis mellifera. PLoS One. 2012;7(1):e30023. doi: 10.1371/journal.pone.0030023.
6. Fischer J, Muller T, Spatz AK, Greggers U, Grunewald B, Menzel R. Neonicotinoids interfere with specific components of navigation in honeybees. PLoS One. 2014;9(3):e91364. doi: 10.1371/journal.pone.0091364.
7. Matsumoto T. Reduction in homing flights in the honey bee Apis mellifera after a sublethal dose of neonicotinoid insecticides. Bull Insect. 2013;66(1):1-9.
8. Williamson SM, Wright GA. Exposure to multiple cholinergic pesticides impairs olfactory learning and memory in honeybees. J Exp Biol. 2013;216(Pt 10):1799-807. doi: 10.1242/jeb.083931.
9. Medrzycki P, Montanari R, Bortolotti L, Sabatini A, Maini S, Porrin C. Effects of imidacloprid administered in sub-lethal doses on honey bee behaviour. Laboratory tests. Bull Insect. 2003; 56(1):59-62.
10. Tan K, Chen W, Dong S, Liu X, Wang Y, Nieh JC. A neonicotinoid impairs olfactory learning in Asian honey bees (Apis cerana) exposed as larvae or as adults. Sci Rep. 2015;5:10989. doi: 10.1038/srep10989.
11. Guez D, Suchail S, Gauthier M, Maleszka R, Belzunces LP. Contrasting effects of imidacloprid on habituation in 7- and 8-day-old honeybees (Apis mellifera). Neurobiol Learn Mem. 2001;76(2):183-91. doi: 10.1006/nlme.2000.3995.
12. Suchail S, Guez D, Belzunces LP. Discrepancy between acute and chronic toxicity induced by imidacloprid and its metabolites in Apis mellifera. Environ Toxicol Chem. 2001;20(11):2482-6.
13. Yang EC, Chuang YC, Chen YL, Chang LH. Abnormal foraging behavior induced by sublethal dosage of imidacloprid in the honey bee (Hymenoptera: Apidae). J Econ Entomol. 2008;101(6):1743-8. doi: 10.1603/0022-0493-101.6.1743.
14. Eiri DM, Nieh JC. A nicotinic acetylcholine receptor agonist affects honey bee sucrose responsiveness and decreases waggle dancing. J Exp Biol. 2012;215(Pt 12):2022-9. doi: 10.1242/jeb.068718.

15. Williamson SM, Willis SJ, Wright GA. Exposure to neonicotinoids influences the motor function of adult worker honeybees. Ecotoxicology. 2014;23(8):1409-18. doi: 10.1007/s10646-014-1283-x.
16. Lambin M, Armengaud C, Raymond S, Gauthier M. Imidacloprid-induced facilitation of the proboscis extension reflex habituation in the honeybee. Arch Insect Biochem Physiol. 2001;48(3):129-34. doi: 10.1002/arch.1065.
17. Charreton M, Decourtye A, Henry M, Rodet G, Sandoz JC, Charnet P, et al. A Locomotor Deficit Induced by Sublethal Doses of Pyrethroid and Neonicotinoid Insecticides in the Honeybee Apis mellifera. PLoS One. 2015;10(12):e0144879. doi: 10.1371/journal.pone.0144879.
18. Oliveira R, Roat T, Carvalho S, O M. Side-effects of thiamethoxam on the brain andmidgut of the africanized honeybee Apis mellifera (Hymenopptera: Apidae). Environ Toxicol. 2014 Oct 29(10):1122-33. Epub Epub 2013 Jan 22. doi: 10.1002/tox.21842.
19. Henry M, Beguin M, Requier F, Rollin O, Odoux JF, Aupinel P, et al. A common pesticide decreases foraging success and survival in honey bees. Science. 2012;336(6079):348-50. doi: 10.1126/science.1215039.
20. Tosi S, Burgio G, Nieh JC. A common neonicotinoid pesticide, thiamethoxam, impairs honey bee flight ability. Sci Rep. 2017;7(1):1201. doi: 10.1038/s41598-017-01361-8.
21. Badiou-Beneteau A, Carvalho SM, Brunet JL, Carvalho GA, Bulete A, Giroud B, et al. Development of biomarkers of exposure to xenobiotics in the honey bee Apis mellifera: application to the systemic insecticide thiamethoxam. Ecotoxicol Environ Saf. 2012;82:22-31. doi: 10.1016/j.ecoenv.2012.05.005.
